# The estrous cycle modulates early-life adversity effects on mouse avoidance behavior through progesterone signaling

**DOI:** 10.1038/s41467-022-35068-w

**Published:** 2022-12-07

**Authors:** Blake J. Laham, Sahana S. Murthy, Monica Hanani, Mona Clappier, Sydney Boyer, Betsy Vasquez, Elizabeth Gould

**Affiliations:** Princeton Neuroscience Institute, Princeton, NJ 08450 USA

**Keywords:** Limbic system, Stress and resilience

## Abstract

Early-life adversity (ELA) increases the likelihood of neuropsychiatric diagnoses, which are more prevalent in women than men. Since changes in reproductive hormone levels can also increase the probability of anxiety disorders in women, we examined the effects of ELA on adult female mice across the estrous cycle. We found that during diestrus, when progesterone levels are relatively high, ELA mice exhibit increased avoidance behavior and increased theta oscillation power in the ventral hippocampus (vHIP). We also found that diestrus ELA mice had higher levels of progesterone and lower levels of allopregnanolone, a neurosteroid metabolite of progesterone, in the vHIP compared with control-reared mice. Progesterone receptor antagonism normalized avoidance behavior in ELA mice, while treatment with a negative allosteric modulator of allopregnanolone promoted avoidance behavior in control mice. These results suggest that altered vHIP progesterone and allopregnanolone signaling during diestrus increases avoidance behavior in ELA mice.

## Abstract (web summary)

Early-life adversity (ELA) can lead to anxiety disorders, which are more prevalent and debilitating in women than men. Here, authors reveal how ELA interacts with neurosteroid synthesis in the hippocampus to drive avoidance behavior in female mice.

## Introduction

Early-life adversity (ELA), which includes childhood maltreatment, chronic illnesses, accidents, natural disasters, and witnessing violence, significantly increases the likelihood of developing many forms of physical and mental illness in adulthood^1–4^. Among these conditions are anxiety disorders, the most prevalent psychiatric disorders^5^. Studies have shown that women are almost twice as likely to have an anxiety disorder diagnosis as men^5–7^ and that anxiety disorders in women are more disabling^8,9^. While sex differences in response to ELA may not be evident during childhood, changes in hormone status are thought to “unmask” vulnerability^10^. Indeed, some women experience increased anxiety during times of dramatic reproductive hormone change, including puberty, pregnancy, childbirth, and menopause (both surgical and age-related)^11–16^, as well as at specific stages of the menstrual cycle^17,18^. Furthermore, childhood maltreatment increases the strength of the association between anxiety disorders and times of hormonal change^19–24^. Taken together, these findings suggest that ELA interacts with ovarian steroids to modulate vulnerability to anxiety disorders. However, the mechanisms that underlie this interaction remain unknown.

Efforts to understand how ELA affects the brain at the cellular and circuit levels in the service of vulnerability have involved the use of multiple animal models. Different mouse models of ELA have been shown to produce different behavioral phenotypes^25–27^, similar to human studies showing that different kinds of childhood maltreatment differentially predispose individuals to certain neuropsychiatric conditions^27–30^. Operationalizing anxiety in the mouse can be problematic given the psychological aspects of anxiety in humans that involve conscious awareness^31^. Less complex symptoms, such as avoidance or behavioral inhibition, as well as restlessness and agitation^32,33^, may be effectively measured in mice by use of standard tests of avoidance behavior and locomotion, respectively. Using the ELA paradigm of maternal separation and early weaning (MSEW) in mice, we and others have found increased avoidance behavior and activity levels compared to control-reared mice^34–36^. These studies have either not tested female mice^34,35^ or found no effect of MSEW on these behaviors in females^36^. The possibility that the estrous cycle may obscure effects of ELA has not yet been investigated.

Studies have shown that avoidance behavior and locomotion in mice, as well as self-reported anxiety in humans, are positively associated with neuronal oscillations in the theta range (4-12 Hz) in the hippocampus^37–40^. In control mice, optogenetic stimulation of ventral hippocampus (vHIP) terminals in the medial prefrontal cortex at theta frequency increases avoidance behavior^41^, and benzodiazepine treatment diminishes avoidance behavior coincident with decreased theta power^42,43^. Parvalbumin-positive (PV + ) interneurons contribute to neuronal oscillations in the hippocampus by coordinating fast inhibition of principal neurons^44^. A subpopulation of PV + interneurons is surrounded by perineuronal nets (PNNs), specialized extracellular matrix structures that are known to regulate plasticity^45^. PNNs have been shown to alter neuronal oscillations^46,47^, raising the possibility that they are involved in the regulation of avoidance behavior. We have shown that MSEW increases PNNs surrounding vHIP PV + cells and increases theta power coincident with increased avoidance behavior and activity levels^36^. However, these studies were not carried out in females, raising questions about whether similar mechanisms might underlie the connection between ELA and behavioral vulnerability.

To investigate whether ovarian status influences the effects of MSEW on behavior, as well as on neuronal oscillations and PNNs in vHIP, we examined control- and MSEW-reared female mice at different stages of estrous, including proestrus, estrus, metestrus, and diestrus, and after ovariectomy. We found that during diestrus, MSEW mice displayed increased avoidance behavior, reduced grooming, and altered locomotion in different contexts. Coincident with these behavioral effects, we observed increased theta power in vHIP when MSEW mice were in diestrus, not estrus. In control mice, the number of PNN + cells in vHIP changed across the estrous cycle, an effect that was prevented in MSEW mice. MSEW-mediated changes in PNN intensity, size, and composition were noted, but only during diestrus. Ovariectomy prevented several MSEW effects, but only in a low stress context, suggesting that MSEW induces an underlying vulnerability that can be partially modulated by ovarian steroids.

The ovaries are the main source of circulating estrogen in females whereas progesterone is produced not only in the ovaries but also in the adrenal glands, where its release is stimulated by stress^48,49^. We confirmed that ovariectomized mice exhibit increased levels of progesterone in both the periphery and vHIP under stressful conditions. We also found that MSEW mice have higher levels of progesterone in vHIP than control-reared mice during diestrus, but not estrus. In addition, MSEW mice have lower levels of the progesterone metabolite allopreganolone than control-reared mice during diestrus, but not estrus. Since elevated progesterone has been shown to increase avoidance in rodents^50–52^, while allopregnanolone generally has the opposite effect^53–55^, we next tested whether manipulating the activation of progesterone receptors or the action of allopregnanolone peripherally or directly in vHIP during diestrus would affect avoidance behavior. We found that progesterone receptor antagonism impeded the increase in avoidance behavior and reduction in grooming in MSEW diestrus mice, while inhibiting allopregnanolone action in control mice mimicked the increase in avoidance behavior of MSEW mice. Taken together with our observations that MSEW diestrus mice have decreased vCA1 expression of steroid 5α-reductase I, an enzyme involved in the local reduction of progesterone to its neurosteroid metabolites, these results suggest that the diminished conversion of progesterone to allopregnanolone in MSEW diestrus mice contributes to increased avoidance behavior.

## Results

### MSEW increases avoidance behavior during diestrus, but not estrus

**Fig. 1:**
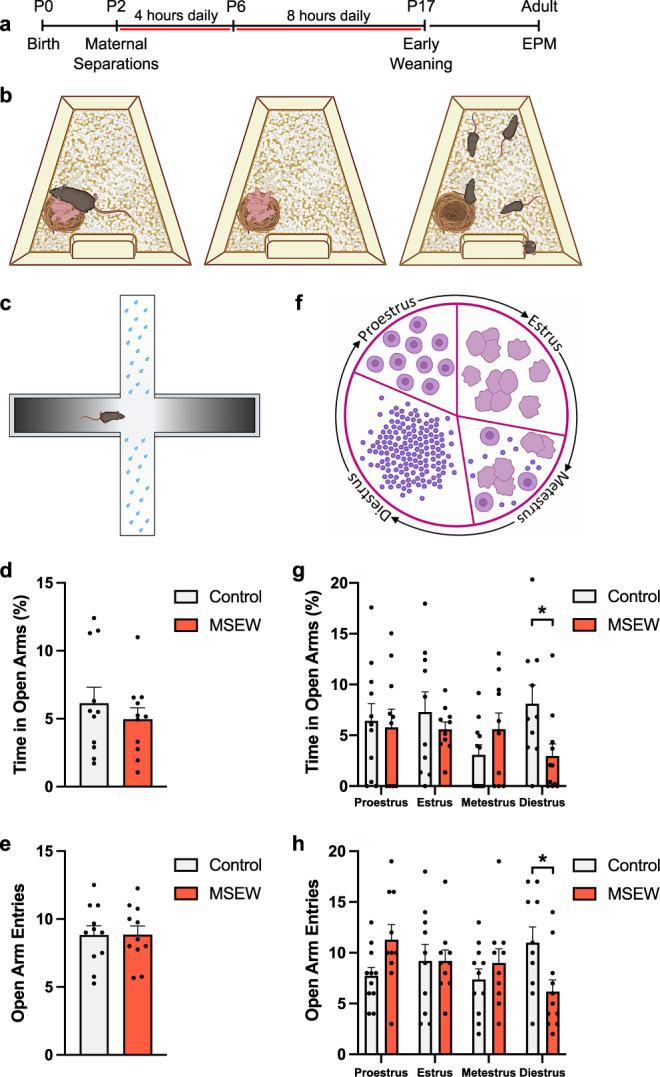
MSEW increases avoidance behavior only in diestrus. **a**, **b** Timeline and schematics of MSEW paradigm. **c** Schematic of the modified EPM, with a fine mist of water sprayed on the open arms to increase avoidance and prevent habituation with repeated trials. **d** Percent time in the open arms of the modified EPM is not significantly different between control and MSEW mice when stage of estrous during testing is not taken into consideration (Control *n* = 11, MSEW *n* = 11). **e** Number of entries into the open arm between control and MSEW mice is not significantly different when stage of estrous during testing is not taken into consideration (Control *n* = 11, MSEW *n* = 11). **f** Schematic of vaginal cytology used to determine whether mice were in different stages of the estrous cycle (proestrus, estrus, metestrus, and diestrus). **g** Testing on the modified EPM at specific stages of the estrous cycle shows that during diestrus, MSEW mice display increased avoidance behavior, i.e., reduced percent time in the open arms, compared to control mice (*F*_3,55_ = 3.393, *p* = 0.0242; Control-MSEW Diestrus *p* = 0.0464; Control *n* = 11, MSEW *n* = 11). **h** MSEW mice also have significantly fewer entries into the open arm while in diestrus (*F*_3,55_ = 4.153, *p* = 0.0101; Control-MSEW Diestrus p = 0.0350; Control *n* = 11, MSEW *n* = 11). No significant differences between control and MSEW mice were observed during proestrus, estrus, or metestrus. MSEW = maternal separation early weaning. *p < 0.05 two-sided unpaired *t*-tests (**d**, **e**); mixed-effects model repeated measures followed by Šidák post hoc tests (**g**, **h**). Data are presented as mean values + SEM (**d**, **e**, **g**, **h**). Images in **b**, **c**, **f** were created using BioRender.com. Source data are provided as a Source Data file.

To determine whether the effects of ELA on avoidance behavior are modulated by estrous cycle stage, we subjected mouse pups to the MSEW paradigm (Fig. 1a, b), followed by testing on the elevated plus maze (EPM) in adulthood during proestrus, estrus, metestrus and diestrus. Since repeated testing on the EPM has been shown to influence avoidance behavior^56^, we modified the task by increasing illumination and spraying a fine mist of water droplets on the open arms. In a pilot study, we tested mice on the “dry EPM” followed by two separate exposures to the “wet EPM”. We found that mice were significantly more avoidant of the open arms on the wet EPM than the dry EPM, and observed no evidence of habituation with repeated testing on the wet EPM (One-way ANOVA *F*_2,15_ = 48.29, *p* = 0.0001, Tukey post hoc test dry vs. wet 1 *p* = 0.0001, dry vs wet 2 *p* = 0.0001, wet 1-wet 2 *p* = 0.8724) (Fig. S1c). Therefore, we continued to use the wet EPM to assess avoidance behavior during each of the four stages of the estrous cycle (Fig. 1c). When the data were combined across stages of the estrous cycle, no differences were observed in percent time spent in the open arms or number of open arm entries between control and MSEW mice (percent time: unpaired t-test t_20_ = 0.8227, *p* = 0.4204; entries: unpaired t-test t_20_ = 0.02448, *p* = 0.9807) (Fig. 1d, e). However, when the data were analyzed considering estrous stage as a variable, a significant interaction was noted between estrous and MSEW (Percent time: Mixed-effects model repeated measures Estrous x MSEW: *F*_3,55_ = 3.393, *p* = 0.0242; entries: Mixed-effects model repeated measures *F*_3,55_ = 4.153, *p* = 0.0101), with a significant increase in avoidance behavior (i.e., a decrease in percent time spent on the open arms and number of open arm entries) between control and MSEW mice during diestrus (Percent time: Šidák post hoc test Control-MSEW Diestrus *p* = 0.0464; Open arm entries: Šidák post hoc test Control-MSEW Diestrus *p* = 0.0350) (Fig. 1g, h).

### MSEW alters activity levels in certain contexts during diestrus, but not estrus

**Fig. 2:**
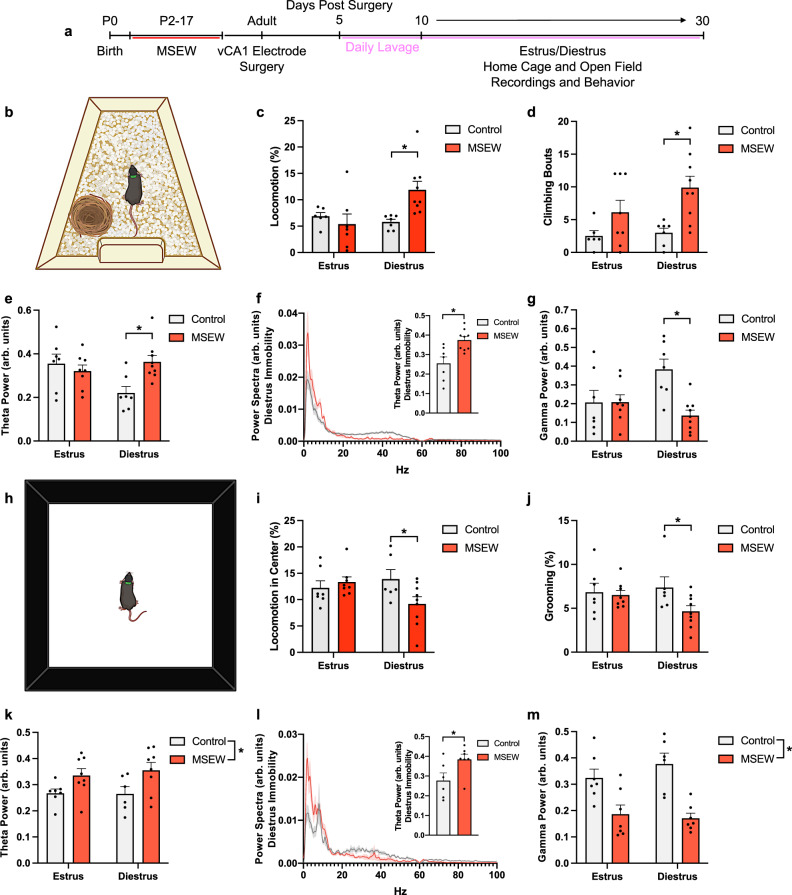
MSEW alters activity levels and vHIP theta oscillations depending on the context. **a** Timeline of experiment. **b** Schematic of recording in home cage. **c** Locomotion in the home cage is increased in MSEW mice in diestrus (*F*_1,8_ = 7.132, *p* = 0.0283; Control-MSEW Diestrus *p* = 0.0226; Control *n* = 9, MSEW *n* = 10). **d** Climbing in the home cage is increased in MSEW mice in diestrus (*F*_1,10_ = 5.112, *p* = 0.0473; Control-MSEW Diestrus *p* = 0.0027; Control *n* = 8, MSEW *n* = 10). **e** vHIP theta power is increased in MSEW mice in diestrus (*F*_1,10_ = 7.238, *p* = 0.0238; Control-MSEW Diestrus *p* = 0.0090; Control *n* = 9, MSEW *n* = 10). **f** Power spectra from diestrus vHIP LFPs during home cage immobility in control and MSEW mice, inset graph shows vHIP theta power is higher in diestrus MSEW mice during immobility (*t*_14_ = 3.384, *p* = 0.0045; Control *n* = 7, MSEW *n* = 9). **g** vHIP gamma power is higher in control diestrus compared to MSEW diestrus (*F*_1,11_ = 11.36, *p* = 0.0063; Control-MSEW Diestrus *p* = 0.0012; Control *n* = 8, MSEW *n* = 10). **h** Schematic of recording in open field. **i** Locomotion in the open field center is reduced in MSEW mice during diestrus (*F*_1,26_ = 4.489, *p* = 0.0438; Control-MSEW Diestrus *p* = 0.0462; Control *n* = 7, MSEW *n* = 10). **j** Grooming in the open field is reduced in MSEW mice during diestrus (*F*_1,11_ = 11.25, *p* = 0.0064; Control-MSEW Diestrus *p* = 0.0450; Control *n* = 7, MSEW *n* = 10). **k** vHIP theta power is higher in the open field in MSEW mice (Estrous: *F*_1,25_ = 9.068, *p* = 0.0059; Control *n* = 7, MSEW *n* = 10). **l** Power spectra from diestrus vHIP LFPs during open field immobility, inset bar graph shows vHIP theta power is higher in diestrus MSEW mice during immobility (*t*_11_ = 2.344, *p* = 0.0389; Control *n* = 6, MSEW *n* = 7). **m** vHIP gamma power is reduced in MSEW mice (Estrous: *F*_1,14_ = 13.09, *p* = 0.0028; Control *n* = 7, MSEW *n* = 9). MSEW = maternal separation early weaning; arb. units = arbitrary units. *p < 0.05, mixed-effects model repeated measures (MSEW x Estrous) followed by Šidák post hoc tests (**c**, **d**, **e**, **g**, **i**, **j**); two-sided unpaired-tests (**f**, **l**). Data are presented as mean values + SEM for error bars (**c**-**g**; **i**-**m**) and ± SEM for bands (**f**, **l**). Images in **b**, **h** were created using BioRender.com. Source data are provided as a Source Data file.

We designed our subsequent studies to compare mice in estrus and diestrus, because these are the two longest estrous cycle phases^57^, with one phase, estrus, showing no significant difference in avoidance behavior between control and MSEW mice, while the other, diestrus, revealed more avoidance behavior in MSEW mice compared to control mice. We first observed behavior of control and MSEW mice in the home cage during estrus and diestrus and found no differences in activity levels (locomotion, climbing) between control and MSEW mice during estrus, but observed more locomotion (Mixed-effects model repeated measures Estrous x MSEW: *F*_1,8_ = 7.132, *p* = 0.0283; Šidák post hoc test Control–MSEW Diestrus *p* = 0.0226) and a greater number of climbing bouts (Mixed-effects model repeated measures Estrous x MSEW *F*_1,10_ = 5.112, *p* = 0.0473, Šidák post hoc test Control-MSEW Diestrus *p* = 0.0027) in MSEW diestrus mice only (Fig. 2c, d).

We also assessed behavior in brightly lit novel environments, which differed in translucence (transparent and opaque) and lighting across trials to minimize habituation. We found no overall changes in locomotion between control and MSEW mice during estrus, but observed decreased locomotion in the center of the open field and increased locomotion in the periphery in MSEW mice during diestrus (Fig. 2i) (Mixed-effects model repeated measures Estrous x MSEW: *F*_1,26_ = 4.489, *p* = 0.0438; Šidák post hoc test Control–MSEW Diestrus *p* = 0.0462). We additionally measured another stress-sensitive behavior, grooming^58–60^, and found no difference between control and MSEW mice while in estrus, but observed decreased grooming in MSEW mice during diestrus (Mixed-effects model repeated measures Estrous x MSEW: *F*_1,11_ = 11.25, *p* = 0.0064, Šidák post hoc test Control-MSEW Diestrus *p* = 0.0450) (Fig. 2j). Taken together, these findings suggest that MSEW diestrus mice display decreased locomotion in the center of the open field and decreased overall grooming, as well as increased activity when in contexts that are likely to be lower threat, i.e., the home cage and periphery of a novel environment.

### MSEW increases theta power in the ventral hippocampus during diestrus, but not estrus

Because the hippocampus has been linked to sex differences in stress effects^61^, we recorded LFPs from the ventral CA1 (vCA1) of control and MSEW female mice during behavioral testing, as vHIP theta power has been linked to avoidant behavior^41^ and is increased in male mice after MSEW^36^. In the home cage, we observed significantly higher oscillatory power in the theta range (4-12 Hz) in MSEW mice while they were in diestrus, but not in estrus, compared to control mice (Mixed-effects model repeated measures Estrous x MSEW: *F*_1,10_ = 7.238, *p* = 0.0238, Šidák post hoc test Control-MSEW Diestrus *p* = 0.0090) (Fig. 2e). Given that MSEW mice in diestrus displayed increased locomotion compared to control mice in diestrus, and because theta oscillations in the dorsal hippocampus (dHIP) have been linked to running^37^, we next investigated if theta power was also increased during periods of immobility by analyzing LFPs during time-stamped behavioral epochs. MSEW mice demonstrated significantly higher vCA1 theta power in diestrus during periods of immobility than control mice (unpaired t-test t_14_ = 3.384, *p* = 0.0045) (Fig. 2f), and a correlational analysis of theta power in vCA1 and locomotion revealed that although there is an overall group effect of increased locomotion and increased theta power in the MSEW diestrus group, increased locomotion is not driving the increase in theta power (Control: *r* = 0.04925, *p* = 0.8812, MSEW: *r* = 0.01204, *p* = 0.7787) (Fig. S2e).

In the open field, MSEW mice also showed an increase in vCA1 theta power compared to control mice (Fig. 2k) (Mixed-effects model repeated measures MSEW *F*_1,25_ = 9.068, *p* = 0.0059). This effect persisted throughout periods of immobility (unpaired t-test t_11_ = 2.344, *p* = 0.0389) (Fig. 2l; Fig. S4), and a correlational analysis of vCA1 theta power and open field locomotion revealed that increased locomotion does not drive increased theta power in the open field (Control: *r* = 0.2023, *p* = 0.3709, MSEW: *r* = 04870, *p* = 0.5683) (Fig. S2j). In both the home cage and open field, MSEW mice in diestrus displayed lower gamma oscillation power compared to control mice in diestrus (Home cage: Mixed-effects model RM Estrous x MSEW: *F*_1,11_ = 11.36, *p* = 0.0063, Šidák post hoc test Control-MSEW Diestrus *p* = 0.0012, MSEW *F*_1,14_ = 5.277, *p* = 0.0354; Open field: Mixed-effects model RM MSEW *F*_1,14_ = 13.09, *p* = 0.0028). No significant change in gamma power was observed in MSEW mice during estrus (Fig. 2g). This finding is in line with previous work showing that chronic stress in adulthood decreases gamma oscillation power within the hippocampus^62^. Given that high-frequency oscillations in vHIP inhibit the basolateral amygdala and reduce freezing in a contextual fear conditioning paradigm^63^, the reduction of vHIP gamma oscillation power in MSEW diestrus mice might coincide with diminished emotional regulation.

### Ovariectomy has a context-dependent influence on behavior and ventral hippocampal theta power in MSEW mice

**Fig. 3:**
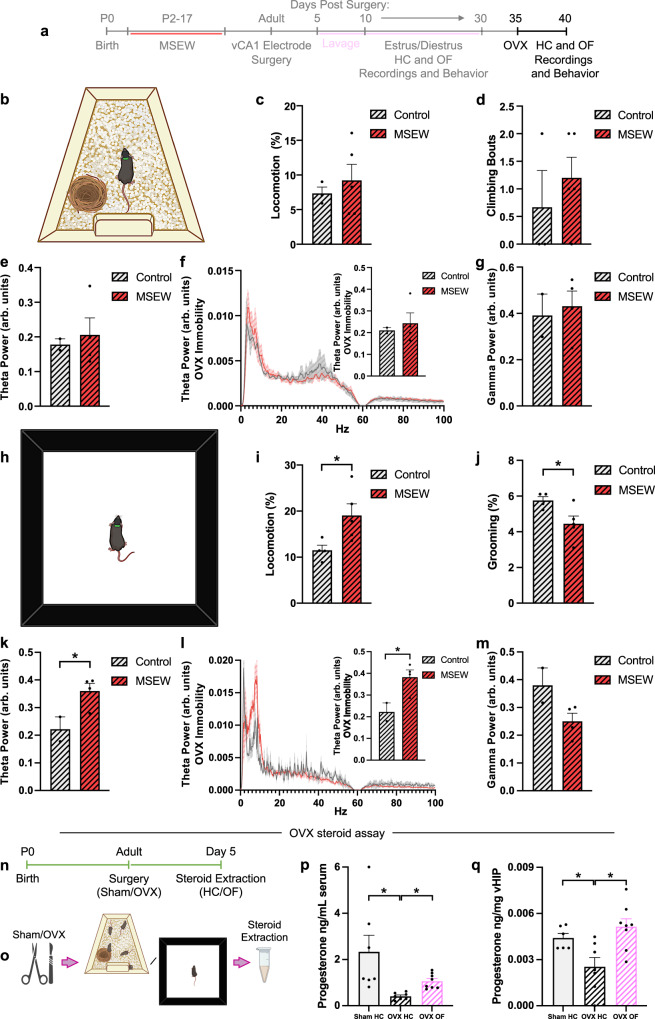
OVX reverses the effects of MSEW seen in diestrus mice, but only in a low stress context. **a** Timeline of experiment. **b** Schematic of recording in the home cage. **c** After OVX, locomotion in the home cage does not differ between groups (Control *n* = 3, MSEW *n* = 5). **d** After OVX, climbing bouts in the home cage do not differ between groups (Control *n* = 3, MSEW *n* = 5). **e** After OVX, vHIP theta power does not differ between groups (Control *n* = 2, MSEW *n* = 4). **f** Power spectra from OVX control and MSEW mice during home cage immobility; inset bar graph shows no difference in vHIP theta power between OVX control and MSEW (Control *n* = 2, MSEW *n* = 4). **g** After OVX, vHIP gamma power in the home cage does not differ between groups (Control *n* = 2, MSEW *n* = 4). **h** Schematic of recording in the open field. **i** After OVX, MSEW exhibit elevated locomotion (*t*_7_ = 2.509, *p* = 0.0405) and **j** decreased grooming in the open field (*t*_7_ = 2.497, *p* = 0.0412) (Control *n* = 4, MSEW *n* = 5). **k** After OVX, vHIP theta power is higher in the open field of MSEW mice (*t*_4_ = 2.791, *p* = 0.0493; Control *n* = 2, MSEW *n* = 4). **l** Power spectra from OVX vHIP LFPs during open field immobility; inset bar graph shows vHIP theta power is higher in OVX MSEW mice (*t*_4_ = 2.866, *p* = 0.0456; Control *n* = 2, MSEW *n* = 4). **m** After OVX, vHIP gamma power in the open field does not differ between groups (Control *n* = 2, MSEW *n* = 4). **n**, **o** Timeline of experiment. **p**, **q** OVX decreases serum and vHIP progesterone levels, but open field exposure raises progesterone levels (Serum: Kruskal-Wallis test *H* = 14.77, *p* < 0.0001; Sham HC-OVX HC *p* = 0.0006, OVX HC-OVX OF *p* = 0.0158; Sham *n* = 7, OVX HC *n* = 7, OVX OF *n* = 8; vHIP: *F*_2,18_ = 7.173, *p* = 0.0051; Sham HC-OVX HC *p* = 0.0463, OVX HC-OVX OF *p* = 0.0047; Sham *n* = 6, OVX HC *n* = 7, OVX OF *n* = 8). Arb. units = arbitrary units; HC = home cage; MSEW = maternal separation early weaning; OF = open field; OVX = ovariectomy; vHIP = ventral hippocampus. *p < 0.05, two-sided unpaired *t*-tests (**c**, **d**, **e**, **f**, **g**, **i**, **j**, **k**, **l**, **m**); one-way ANOVA with Tukey post hoc tests (**q**). Data are presented as mean values + SEM for error bars (**c-g; i-m, p, q**) and ± SEM for bands (**f, l**). Images in b,h,o were created using BioRender.com. Source data are provided as a Source Data file.

To determine whether differences in behavior and neuronal oscillations observed during diestrus in MSEW mice are dependent on ovarian steroids, control and MSEW mice were bilaterally ovariectomized (OVX) and, after recovery from surgery, tested in the home cage and open field again. After OVX, MSEW mice spend more time moving in the open field (unpaired t-test t_7_ = 2.509, *p* = 0.0405) compared to OVX control mice, and exhibit less grooming (unpaired t-test t_7_ = 2.497, *p* = 0.0412) (Fig. 3i, j). Coincident with increased movement, there was an increase in theta power in vCA1 of OVX MSEW mice compared to OVX control mice (unpaired t-test t_4_ = 2.791, *p* = 0.0493) (Fig. 3k, l), with no significant difference in gamma power (unpaired t-test t_5_ = 0.7466, *p* = 0.4889) (Fig. 3m, S3). Increased vCA1 theta power was observed during time-stamped bouts of immobility in OVX MSEW mice (unpaired t-test t_4_ = 2.866, *p* = 0.0456), ruling out a locomotor-driven increase in theta power. By contrast, no change in locomotion or oscillatory power was observed in OVX MSEW mice in the home cage (Locomotion: unpaired t-test t_6_ = 20.5979, *p* = 0.5718; Climbing: unpaired t-test t_6_ = 0.7651, *p* = 0.4732; Theta power: unpaired t-test t_4_ = 0.3754, *p* = 0.7264; Theta immobility: unpaired t-test t_4_ = 0.4554, *p* = 0.6725; Gamma power: unpaired t-test t_4_ = 0.3509, *p* = 0.7434) (Fig. 3c, d, e, f, g, S3). Collectively, these findings suggest that ovarian steroids are necessary for diestrus MSEW effects, but only in certain contexts. When OVX MSEW mice are in the familiar low-threat home cage environment, their behavior and neuronal oscillations are similar to OVX control mice. However, in a novel, brightly lit open field, locomotion and vCA1 theta oscillations are higher, similar to what is observed with MSEW diestrus mice. The time point we examined after OVX (5 days) was sufficient to reduce but not completely eliminate circulating levels of progesterone as well as circulating levels of the progesterone metabolite allopregnanolone (Fig. 3). We also found that progesterone levels in both the serum and vHIP were increased in OVX mice exposed to a novel environment compared to the relatively stress-free home cage (Serum: Kruskal-Wallis test *H* = 14.77, *p* < 0.0001, Dunn post hoc test: Sham HC-OVX HC *p* = 0.0006, Sham HC-OVX OF *p* = 0.9004, OVX HC-OVX OF *p* = 0158; vHIP: One-way ANOVA *F*_2,18_ = 7.173, *p* = 0.0051, Tukey post hoc test: Sham HC-OVX HC *p* = 0.0463, Sham HC-OVX OF *p* = 0.3258, OVX HC-OVX OF *p* = 0.0047) (Fig. 3p, q). These findings are consistent with previous reports demonstrating that stress increases peripheral progesterone levels after OVX, presumably by stimulating the release of adrenal-synthesized hormones^48,49^.

### Estrous cycle-mediated changes in ventral hippocampal PNNs are disrupted by MSEW and are partially reversed after OVX

PNNs surrounding PV + interneurons have been linked to neuronal oscillations^46,47^ and are increased in the male mouse vHIP after MSEW^36^. Recent studies suggest that PNNs change alongside the diurnal rhythm^64^, raising the possibility that PNNs undergo additional change across the estrous cycle. To investigate this possibility, we perfused control and MSEW mice at the same time of day and examined PNNs across the estrous cycle.

**Fig. 4:**
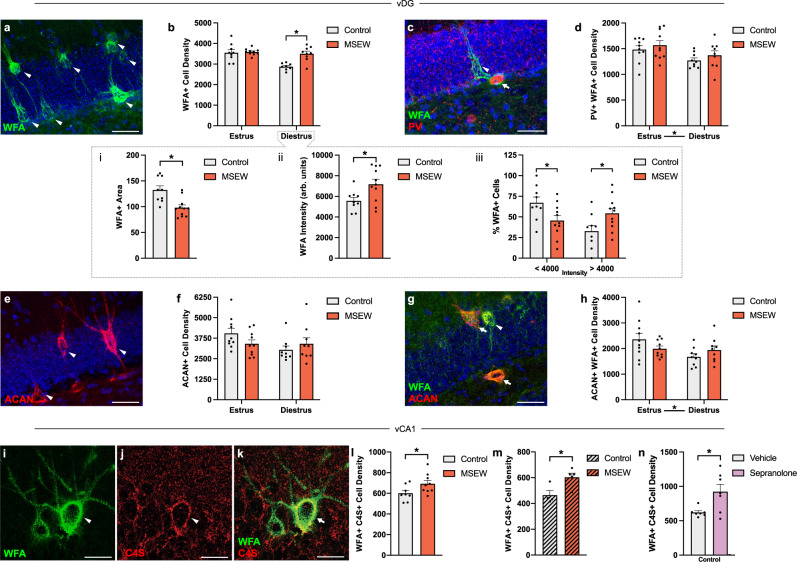
MSEW eliminates estrous cycle-mediated changes in the number of neurons with PNNs and alters PNN intensity and composition. **a** Confocal image of WFA + cells in vDG. **b** The density of WFA + cells is higher during estrus than diestrus in controls (*F*_1,32_ = 14.26, *p* = 0.0007; Control-MSEW Diestrus *p* = 0.0003), but not in MSEW mice (Control: Estrus *n* = 9, Diestrus *n* = 8; MSEW: Estrus *n* = 10, Diestrus *n* = 9). **bi** During diestrus, MSEW mice have smaller PNNs than controls (*t*_17_ = 3.641, *p* = 0.0019; Control *n* = 9, MSEW *n* = 11). **bii** During diestrus, MSEW mice have higher WFA + intensities than controls (*t*_17_ = 2.562, *p* = 0.0196; Control *n* = 9, MSEW *n* = 11). **biii** During diestrus, control and MSEW WFA + PNNs differ in intensity (*F*_1,18_ = 5.455, *p* = 0.0313; <4000 *p* = 0.0498; >4000 *p* = 0.0498; Control *n* = 9, MSEW *n* = 11). **c** Confocal image of PV + /WFA + cells in vDG. **d** The density of WFA + /PV + cells changes across the estrous cycle (*F*_1,34_ = 7.001, *p* = 0.0123; Control: Estrus *n* = 10, Diestrus *n* = 9; MSEW: Estrus *n* = 10, Diestrus: *n* = 9). **e** Confocal image of ACAN + cells in vDG. **f** ACAN + cell density is unchanged across the estrous cycle (Control *n* = 10, MSEW *n* = 10). **g** Confocal image of WFA + /ACAN + cells in vDG. **h** WFA + /ACAN + cell density changes across the estrous cycle (Estrous:*F*_1,34_ = 4.817, *p* = 0.0344; Control: Estrus *n* = 10, Diestrus *n* = 9; MSEW: Estrus *n* = 10, Diestrus: *n* = 9). **i**, **j**, **k** Confocal images of WFA + cells in vCA1, one is positive for C4S. **l** During diestrus, MSEW mice have a higher WFA + /C4S + cell density than controls (*t*_15_ = 2.178, *p* = 0.0458; Control *n* = 8, MSEW *n* = 9). **m** After OVX, MSEW mice have higher WFA + /C4S + cell density than controls (*t*_7_ = 2.953, *p* = 0.0213; Control *n* = 4, MSEW *n* = 5). **n** Sepranolone administration increases the density of WFA + /C4S + cell density (*t*_12_ = 2.755, *p* = 0.0174; Control *n* = 7, MSEW *n* = 7). ACAN = aggrecan; arb. units = arbitrary units; C4S = chondroitin 4-sulfation; MSEW = maternal separation early weaning; PNN = perineuronal net; PV = parvalbumin; vDG = ventral dentate gyrus; WFA = wisteria floribunda agglutinin. *p < 0.05, two-way ANOVA followed by Šidák tests (**b**, **biii**, **d**, **f**, **h**); two-sided unpaired *t*-tests (**bi**, **bii**, **l**, **m**, **n**). Scale bars: 40 μm (**a**, **c**, **e**, **g**); 20 μm (**I**, **j**, **k**). Data are presented as mean values + SEM (**b, bi, bii, biii, d, f, h, l, m, n**). Source data are provided as a Source Data file.

In the ventral dentate gyrus (vDG), we found estrous cycle differences in the number of cells labeled with the lectin-based PNN marker *wisteria floribunda agglutinin* (WFA) (Two-way ANOVA Estrous x MSEW: *F*_1,32_ = 14.26, *p* = 0.0007), with more cells observed during estrus than during diestrus (Šidák post hoc test Control-MSEW: Diestrus *p* = 0.0003) (Fig. 4a, b). An additional estrous cycle effect was observed when examining the number of PV + cells with PNNs (Two-way ANOVA Estrous: *F*_1,34_ = 7.001, *p* = 0.0123), although no differences were observed between diestrus and estrus, control or MSEW in the number of PV + cells (Two-way ANOVA Estrous: *F*_1,32_ = 3.025, *p* = 0.916) (Fig. S7a). Because a subset of basket cells are CCK + , we examined this population and found no effects of estrous cycle or MSEW on cell density (Two-way ANOVA Estrous: *F*_1,21_ = 2.676, *p* = 0.1168) (Fig. S7b).

To explore the influence of the estrous cycle on PNNs in more depth, we also examined the main chondroitin sulfate proteoglycan (CSPG) aggrecan (ACAN). We found estrous cycle differences in ACAN + WFA + cell density (Two-way ANOVA Estrous: *F*_1,34_ = 4.817, *p* = 0.0344, Estrous x MSEW: *F*_1,34_ = 3.598, *p* = 0.0664) with higher cell density in estrus compared to diestrus (Šidák post hoc test Estrus-Diestrus Control *p* = 0.0130), but no estrous cycle differences in ACAN + cell densities (Two-way ANOVA Estrous x MSEW: *F*_1,34_ = 3.031, *p* = 0.0907, Šidák post hoc test Estrus-Diestrus Control: *p* = 0.0377) (Fig. 4e, f, g, h). In MSEW mice, estrous cycle differences in vDG PNNs were not evident in WFA + cells (*p* = 0.7881), PV + WFA + cells (*p* = 0.1523), ACAN + cells (*p* = 0.999), or ACAN + WFA + cells (*p* = 0.9709) (Šidák post hoc test). In vDG, no significant differences were observed in the percentage of PV + cells that were WFA + , WFA + cells that were PV + , ACAN + cells that were WFA + , or WFA + cells that were ACAN + between diestrus and estrus, or between control and MSEW (Table S1). These findings suggest that naturally occurring plasticity of PNNs surrounding PV + cells across the estrous cycle is eliminated in MSEW mice. In vCA1, estrous cycle or MSEW differences were not observed in the numbers or percentages of WFA + (Two-way ANOVA Estrous: *F*_1,34_ = 1.684, *p* = 0.2031), PV + WFA + (Two-way ANOVA Estrous: *F*_1,34_ = 0.009523, *p* = 0.9228) (Fig. S5a, b, c, d), ACAN + WFA + (Two-way ANOVA Estrous: *F*_1,34_ = 0.3683, *p* = 0.5480) (Fig. S5g, h), PV + (Two-way ANOVA Estrous: *F*_1,32_ = 0.3654, *p* = 0.5498), or CCK + cells (Two-way ANOVA Estrous: *F*_1,34_ = 0.5382, *p* = 0.4682) (Fig. S7c, d; Table S2) However, vCA1 ACAN + density counts revealed a significant interaction (Two-way ANOVA Estrous x MSEW: *F*_1,34_ = 6.534, *p* = 0.0152, Šidák post hoc test Control-MSEW Diestrus *p* = 0.0556) (Fig. S5e, f).

Building off our findings that MSEW behavioral and vHIP oscillation effects are present only during diestrus, we investigated PNNs in control and MSEW diestrus mice in more detail. In vCA1, we found differences in the size of PNNs surrounding PV + cells. MSEW diestrus mice had smaller PNN cross-sectional areas than estrus mice (unpaired t-test t_17_ = 4.636, *p* = 0.0002), an effect that was also observed in vDG (unpaired t-test t_18_ = 3.641, *p* = 0.0019) (Fig. 4bi; S5bi). We observed no changes in PV + area in vDG (unpaired t-test t_16_ = 1.026, *p* = 0.3203) or vCA1 (unpaired t-test t_13_ = 0.04777, *p* = 0.9626) (Fig. S6a, g). In vDG, we also found increased overall intensity of PNNs in MSEW diestrus mice compared to controls (Two-way ANOVA Estrous x MSEW: *F*_1,18_ = 5.455, *p* = 0.0313, Šidák post hoc test Control-MSEW < 4000: *p* = 0.0498; > 4000 *p* = 0.0498), due to an apparent shift toward more cells in the most intense PNN category in the overall population (Fig. 4bii, biii). This effect was not observed in vCA1 (Two-way ANOVA Estrous x MSEW: *F*_1,17_ = 0.1149, *p* = 0.7388; Estrous: *F*_1,17_ = 506.5, *p* < 0.0001) (Fig. S5bii, biii).

Differences in PNN composition can influence neuronal function, leading us to examine the expression of the 4-sulfation pattern of chondroitin sulfate chains, which has been associated with reduced plasticity in PNNs^65,66^. We found significant increases in C4S + WFA + cells in vCA1 (Fig. 4i, j, k, l) (unpaired t-test t_15_ = 2.178, *p* = 0.0458) of MSEW diestrus mice compared to control diestrus mice. These findings indicate that in vCA1, MSEW mice in diestrus have smaller PNNs with more PNNs containing C4S compared to control mice. After OVX, differences observed between control and MSEW diestrus mice were no longer evident for WFA + cell density (unpaired t-test t_7_ = 0.5536, *p* = 0.5971) or WFA + area (unpaired t-test t_7_ = 1.070, *p* = 0.3201) (Fig. S6b, d), but C4S + WFA + cell density in vCA1 remained higher in MSEW compared to control mice (unpaired t-test t_7_ = 2.953, *p* = 0.0213) (Fig. 4m).

### Progesterone and allopregnanolone signaling in the ventral hippocampus are altered by MSEW and influence avoidance behavior during diestrus

**Fig. 5:**
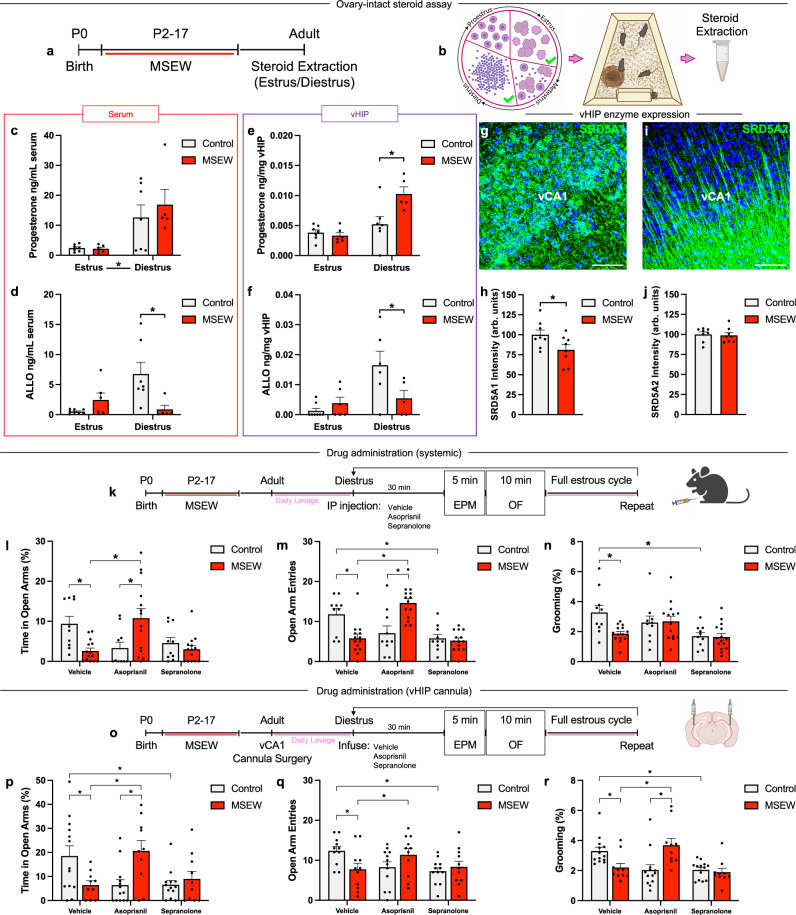
MSEW alters serum and vHIP progesterone and allopregnanolone concentrations. **a**, **b** Timeline and schematic of experiment. **c** Control and MSEW mice have higher serum progesterone in diestrus than estrus (*F*_1,21_ = 15.78, *p* = 0.0007; Control: Estrus *n* = 7, Diestrus *n* = 7; MSEW: Estrus *n* = 6, Diestrus *n* = 5). **d** MSEW mice have lower serum allopregnanolone than diestrus controls (*F*_1,22_ = 10.49, *p* = 0.0038; Control-MSEW Diestrus *p* = 0.0064; Control: Estrus *n* = 7, Diestrus *n* = 7; MSEW: Estrus *n* = 6, Diestrus *n* = 5). **e** MSEW mice have higher vHIP progesterone than controls in diestrus (*F*_1,21_ = 8.846, *p* = 0.0072; Control-MSEW Diestrus *p* = 0.024; Control: Estrus *n* = 7, Diestrus *n* = 7; MSEW: Estrus *n* = 6, Diestrus *n* = 5). **f** Controls have higher vHIP allopregnanolone than diestrus MSEW mice (*F*_1,21_ = 6.320, *p* = 0.0202, Control-MSEW Diestrus *p* = 0.0250; Control: Estrus *n* = 8, Diestrus *n* = 6; MSEW: Estrus *n* = 6, Diestrus *n* = 5). **g** vCA1 SRD5A1 labeling. **h** Diestrus MSEW mice exhibit lower SRD5A1 than controls (*t*_14_ = 2.162, *p* = 0.0484; Control *n* = 8, MSEW *n* = 8). **i** vCA1 SRD5A2 labeling. **j** No difference in vCA1 SRD5A2 (Control *n* = 8, MSEW *n* = 8). **k** Systemic drug administration timeline. Asoprisnil promotes control-like open arm percent time (*F*_2,44_ = 13.00, *p* < 0.0001; Vehicle-Asoprisnil MSEW *p* = 0.0167) **l**, open arm entries (*F*_2,44_ = 18.82, *p* < 0.0001; Vehicle-Asoprisnil MSEW *p* < 0.0001) **m**, and grooming (*F*_2,26_ = 4.086, *p* = 0.0286; Control-MSEW Asoprisnil *p* = 0.9820) **n** in MSEW mice (Control *n* = 10, MSEW *n* = 14). Sepranolone promotes MSEW-like open arm entries (Vehicle-Sepranolone Control *p* = 0.0029) **m** and grooming (Vehicle-Sepranolone Control *p* = 0.0044) **n** in control mice (Control *n* = 10, MSEW *n* = 14). **o** Cannula infusion timeline. vCA1 infusion of asoprisnil promotes control-like percent open arm time (*F*_2,44_ = 18.26, *p* < 0.0001; Vehicle-Asoprisnil MSEW *p* = 0.0003) **p**, open arm entries (*F*_2,41_ = 9.405, *p* = 0.0009; Vehicle-Asoprisnil MSEW *p* = 0.0425) **q**, and grooming (*F*_2,42_ = 14.35, *p* < 0.0001; Vehicle-Asoprisnil MSEW *p* = 0.0014) **r** in MSEW mice. Sepranolone infusion promotes MSEW-like percent open arm time (Vehicle-Sepranolone Control *p* = 0.0005) **p**, open arm entries (Vehicle-Sepranolone Control *p* = 0.0016) **q**, and grooming (Vehicle-Sepranolone Control *p* = 0.0022) **r** in controls (Control *n* = 13, MSEW *n* = 10). ALLO = allopregnanolone; Arb. units = arbitrary units; EPM = elevated plus maze; MSEW = maternal separation early weaning; OF = open field; SRD5A1 = steroid 5α-reductase I; SRD5A2 = steroid 5α-reductase 2; vCA1 = ventral CA1; vHIP = ventral hippocampus. *p < 0.05, two-way ANOVA followed by Šidák tests (**c**, **d**, **e**, **f**); two-tailed unpaired *t*-test (**h**, **j**); two-way repeated measures ANOVA with Šidák tests (**l**, **m**, **n**, **p**, **q**, **r**). Data are presented as mean values + SEM (**c-f, j, j, l-n, p-r**). Scale bars: 100 μm (**a**, **c**, **e**, **g**). Images in **b**, **k**, **o** were created using BioRender.com. Source data are provided as a Source Data file.

Studies have shown that the level of progesterone is relatively high during diestrus compared to estrus, as well as after ovariectomy under conditions of stress^48,67^, findings we have verified in this report (Figs. 3p, q, 5c). Since progesterone and its metabolite allopregnanolone are known to influence avoidance behavior^50–55^, we next investigated whether MSEW affects ovarian steroids and allopregnanolone levels of females with intact ovaries. We found that during diestrus, MSEW mice had elevated levels of progesterone in vHIP, but not in serum, compared to diestrus control-reared mice (Serum: Two-way ANOVA Estrous: *F*_1,21_ = 15.78, *p* = 0.0007; vHIP: Two-way ANOVA Estrous x MSEW: *F*_1,21_ = 8.846, *p* = 0.0072, Šidák post hoc test Control-MSEW Diestrus *p* = 0.024). MSEW mice also had diminished levels of the neurosteroid allopregnanolone in both serum and vHIP (Serum: Two-way ANOVA Estrous x MSEW *F*_1,22_ = 10.49, *p* = 0.0038, Šidák post hoc test Control-MSEW Diestrus *p* = 0.0064; vHIP: Two-way ANOVA Estrous x MSEW: *F*_1,21_ = 6.320, *p* = 0.0202, Šidák post hoc test Control-MSEW Diestrus *p* = 0.0250) (Fig. 5c, d, e, f). Taken together, these findings suggest reduced conversion of progesterone to allopregnanolone in MSEW mice relative to control mice.

We next investigated the expression of steroid 5α-reductase type I and II in vHIP, enzymes that facilitate the first of two steps in the metabolism of progesterone to allopregnanolone^68^. Previous work has identified diminished 5α-reductase expression in individuals who experienced ELA^69^. We observed a significant decrease in vCA1 steroid 5α-reductase I (SRD5A1) optical intensity in diestrus MSEW mice compared to diestrus control mice (unpaired t-test t_14_ = 2.162, *p* = 0.0484) (Fig. 5g, h). No difference was observed in steroid 5α-reductase II (SRD5A2) intensity (unpaired t-test t_14_ = 0.2431, *p* = 0.8114) (Fig. 5i, j).

We then sought to investigate whether blockade of progesterone and its metabolite allopregnanolone influence avoidance behavior in females during diestrus. Diestrus mice were administered either vehicle, asoprisnil: a selective progesterone receptor modulator with primarily antagonistic action^70^, or sepranolone: a negative allosteric modulator of allopregnanolone’s GABA_A_ receptor binding site^71^, systemically before being tested on the EPM and the open field. After vehicle administration, MSEW diestrus mice spent significantly less time in the open arms than control mice (Two-way repeated measures ANOVA Drug x MSEW: *F*_2,44_ = 13.00, *p* < 0.0001; Šidák post hoc test Control–MSEW Vehicle *p* = 0.0163) (Fig. 5l). After asoprisnil administration, MSEW diestrus mice spent significantly more time in the open arms compared to their vehicle trial (Šidák post hoc test Vehicle-Asoprisnil MSEW *p* = 0.0167), while control diestrus mice spent significantly less time in the open arms compared to their vehicle trial (Šidák post hoc test Vehicle-Asoprisnil Control *p* = 0.041) (Fig. 5l). Taken together, MSEW diestrus mice spent significantly more time in the open arms than control diestrus mice after asoprisnil administration (Šidák post hoc test Control–MSEW Asoprisnil *p* = 0.0440) (Fig. 5l). Sepranolone administration eliminated the difference in time spent in the open arms between control and MSEW mice (Šidák post hoc test Control–MSEW Sepranolone *p* = 0.7305) (Fig. 5l). Entries into the open arms closely mirrored time spent in the open arms (Two-way repeated measures ANOVA Drug x MSEW: *F*_2,44_ = 18.82, *p* < 0.0001; Šidák post hoc test: Control–MSEW Vehicle *p* = 0.0018, Control–MSEW Asoprisnil *p* < 0.0001, Vehicle–Asoprisnil Control *p* = 0.0246, Vehicle–Sepranolone Control *p* = 0.0029, Vehicle–Asoprisnil MSEW *p* < 0.0001, Asoprisnil-Sepranolone MSEW *p* < 0.0001).

We next investigated grooming behavior in the open field after drug administration. Vehicle administration revealed that MSEW diestrus mice spent significantly less time grooming than control diestrus mice in the open field (Two-way repeated measures ANOVA Drug x MSEW: *F*_2,26_ = 4.086, *p* = 0.0286; Šidák post hoc test Control–MSEW Vehicle *p* = 0.0068) (Fig. 5n). Administration of either asoprisnil or sepranolone abolished the difference in grooming time between control and MSEW mice (Šidák post hoc test Control-MSEW Asoprisnil *p* = 0.9820, Control-MSEW Sepranolone *p* = 0.9988) (Fig. 5n). In control diestrus mice, sepranolone administration produced a significant decrease in time spent grooming when compared with their vehicle trial times (Šidák post hoc test Vehicle-Sepranolone Control *p* = 0.0044) (Fig. 5n).

In an additional experiment, we sought to understand whether steroid signaling, in particular allopregnanolone, influences C4S expression within vHIP. Control diestrus mice were administered with an IP injection of either vehicle or sepranolone, an inhibitor of allopregnanolone action. Sepranolone administration significantly increased C4S density in vCA1 compared to vehicle trials (unpaired t-test t_12_ = 2.755, *p* = 0.0174) (Fig. 4n), suggesting that steroid action can dynamically modify PNN composition on a rapid timescale.

Finally, because progesterone receptors and GABA_A_ receptors are present throughout the brain, we sought to determine whether manipulating the action of progesterone or allopregnanolone specifically in vHIP would have similar effects on avoidance behavior as those we observed when using systemic manipulations. Using a similar experimental design as described above, we infused vehicle, asoprisnil, or sepranolone through bilateral vHIP cannula when control and MSEW mice were in diestrus and found similar results to systemic administration. Blocking progesterone receptors diminished avoidance behavior in MSEW diestrus mice, while inhibiting allopregnanolone increased avoidance behavior in control diestrus mice (Two-way repeated measures ANOVA Drug x MSEW: *F*_2,44_ = 18.26, *p* < 0.0001; Šidák post hoc test Control–MSEW Vehicle *p* = 0.0250, Control–MSEW Asoprisnil *p* = 0.0064, Vehicle–Asoprisnil Control *p* = 0.0004, Vehicle–Sepranolone Control *p* = 0.0005, Vehicle–Asoprisnil MSEW *p* = 0.0003, Asoprisnil–Sepranolone MSEW *p* = 0.0028) (Fig. 5p). Local infusion produced similar effects in open arm entries (Two-way repeated measures ANOVA Drug x MSEW: *F*_2,41_ = 9.405, *p* = 0.0009; Šidák post hoc test Control–MSEW Vehicle *p* = 0.0380, Vehicle–Asoprisnil Control *p* = 0.0122, Vehicle–Sepranolone Control *p* = 0.0016, Vehicle–Asoprisnil MSEW *p* = 0.0425) (Fig. 5q), as well as grooming (Two-way repeated measures ANOVA Drug x MSEW: *F*_2,42_ = 14.35, *p* < 0.0001; Šidák post hoc test Control–MSEW Vehicle *p* = 0.0349, Control–MSEW Asoprisnil *p* = 0.0007, Vehicle–Asoprisnil Control *p* = 0.0021, Vehicle–Sepranolone Control *p* = 0.0022, Vehicle–Asoprisnil MSEW *p* = 0.0014, Asoprisnil–Sepranolone MSEW *p* = 0.0001) (Fig. 5r). These results suggest that the modulation of avoidance behavior by progesterone and allopregnanolone signaling in MSEW and control mice can be mediated by vHIP.

Peripheral and central antagonism of progesterone receptors with asoprisnil paradoxically increased avoidance behavior in control-reared diestrus mice. Studies in humans and rodents have shown that progesterone withdrawal, as well as high levels of allopregnanolone, can induce a state of increased anxiety^72,73^. Our findings using asoprisnil in control-reared mice may mimic a steroid profile that resembles these states.

## Discussion

Our findings suggest that the adverse effects of MSEW may be unmasked in females during diestrus. We found that mice subjected to MSEW exhibited fluctuating levels of avoidance behavior on the EPM, with a significant increase in open-arm avoidance during diestrus compared to control mice. No differences in avoidance behavior were observed between control and MSEW mice in other stages of the estrous cycle, including proestrus, estrus, or metestrus. We also found that during diestrus, MSEW mice showed increased activity levels in the home cage (locomotion, climbing), along with decreased locomotion in the center of the open field and decreased grooming, compared to control mice. Similar to what we observed for MSEW male mice^35^, behavioral differences between control and MSEW female mice in diestrus were accompanied by higher vHIP theta power in MSEW mice, including during periods of immobility, as well as alterations in vHIP PNNs. In contrast, none of these differences were observed between control and MSEW mice when they were in estrus.

These findings suggest an interaction between fluctuations in circulating ovarian hormones and MSEW effects. To test this relationship, after MSEW or control rearing, we subjected adult female mice to ovariectomy to eliminate the estrous cycle. Surprisingly, we found that several of the adult behavioral and electrophysiological effects of MSEW persisted. While ovariectomy in adulthood substantially lowers levels of progesterone, progesterone production via the adrenal glands remains intact and is stress sensitive, with adult stress elevating peripheral levels of progesterone^48,49,74^, an effect we replicated in our study. We further found that these differences were even greater in vHIP where stress restored progesterone to sham-operated levels in ovariectomized mice. Thus, stress-induced increases in progesterone levels in adult OVX mice might explain the persistence of diestrus-like behavior and increased vCA1 theta power in MSEW OVX mice when in the novel open field, a potentially stressful environment, but not the home cage. It should also be noted that ovariectomy is known to have multiple actions on the hippocampus, including on dendritic spines, synapses, and the number of inhibitory interneurons^75,76^, which may contribute to some of our observed behavioral, electrophysiological, and histological effects.

Previous studies have shown that PV + interneurons play an important role in the generation of theta oscillations^44^, and that these cells are altered by MSEW in male mice^36^. Most PV + interneurons are surrounded by PNNs, which are also influenced by MSEW in vDG of males^36^, and have been shown in other systems to affect neuronal oscillations^46,47^. Our findings suggest that PNNs change across the estrous cycle in control-reared mice and that this plasticity is disrupted after MSEW. Estrous-mediated plasticity in PNNs may buffer the hippocampus against adverse behavioral effects potentially arising from fluctuations in ovarian steroids. In the absence of this plasticity after MSEW, times of a positive progesterone:estrogen ratio may result in increased avoidance behavior, altered activity levels, and reduced grooming. Although the effects of progesterone on neuronal oscillations have not been well studied in experimental animals, studies in humans have shown a positive association between progesterone and theta power in other brain regions^77^. Since reduced PNNs have been associated with diminished theta^78^, the estrus-diestrus decrease in PNNs observed in controls may compensate for naturally occurring changes in progesterone levels in order to stabilize avoidance behavior across the estrous cycle. In addition to reduced estrous-mediated plasticity after MSEW, we observed reduced PNN size and an increased percentage of PNNs containing a chondroitin-4-sulfation pattern, a PNN constituent associated with reduced plasticity^65,66,79^, in vCA1. The latter finding was also observed in OVX mice between control and MSEW groups, raising the possibility of potential causal links among C4S + PNNs, increased theta oscillations, and alterations in behavior. Future studies will be necessary to test these possibilities directly.

Progesterone levels rise during diestrus, yielding a higher progesterone:estrogen ratio than during estrus^67^. Studies have shown that experimentally elevated progesterone levels can increase avoidance behavior in female mice by binding to progesterone receptors in the hippocampus^50–52^, although many studies have reported that naturally occurring increases in progesterone levels across the estrous cycle do not have this effect^80–82^. Previous studies suggest that ELA does not have a major impact on the estrous cycle or on circulating serum levels of estrogen in adult females^83,84^, raising the possibility that MSEW effects in diestrus may be primarily driven by different brain responses to changing levels of hormones instead of differences in circulating hormone levels themselves. These findings are consistent with human studies showing that excessive anxiety can emerge during times of ovarian steroid change, despite the fact that no clear correlations between anxiety and hormone levels exist^10,85,86^.

In the healthy hippocampus, progesterone is metabolized to the neurosteroid allopregnanolone through two enzymes made by principal neurons^68^. Allopregnanolone is known to bind to GABA_A_ receptors in the hippocampus where it leads to reductions in avoidance and other defensive behaviors^53–55^. In the adult rodent brain, allopregnanolone levels and GABA_A_ receptor density are both modulated across the estrous cycle^87–91^, with higher rates of conversion to allopregnanolone and binding of allopregnanolone to GABA_A_ receptors during diestrus than estrus^90^. These findings suggest that a buffering mechanism exists in the healthy brain to protect against potentially dysfunctional avoidance responses to natural increases in progesterone.

Studies have also shown that ELA reduces both allopregnanolone levels and GABA_A_ receptor binding^92,93^, suggesting that this endogenous buffering mechanism may be disrupted after MSEW, although previous studies have not considered ELA-induced effects on these measures in the context of estrous stage. Our findings are consistent with the possibility that MSEW-induced increases in avoidance behavior during diestrus are the result of diminished conversion of progesterone to allopregnanolone. First, we found that MSEW mice had higher progesterone and lower allopregnanolone levels in vHIP than control-reared mice when in diestrus but not estrus. Second, we observed a reduction in expression of 5α-reductase, an enzyme important for conversion of progesterone to allopregnanolone in vHIP of MSEW mice. Third, we found that treatment with the selective progesterone receptor modulator asoprisnil blocked MSEW-induced increases in avoidance behavior, while treatment with sepranolone, an inhibitor of allopregnanolone action, increased avoidance behavior in control mice. Similar findings were observed whether the drugs were administered systemically or directly into vHIP. Taken together, these data suggest that elevated progesterone levels during diestrus produce increased avoidance behavior in MSEW mice due to an imbalance in the activation of progesterone receptors versus allopregnanolone (GABA_A_) receptors.

Studies have revealed that Holocaust survivors exhibit reduced 5α-reductase I expression, and that the most robust reductions were present in individuals that were youngest at the time of the war^69^. Furthermore, studies investigating postmortem brain tissue reveal that individuals with a major depressive disorder diagnosis exhibit diminished 5α-reductase I expression^94^. This diminished expression was not present in individuals who were receiving antidepressant treatment at the time of death. Additional studies have shown that allopregnanolone is not only modulated across the menstrual cycle^95^, but is reduced in women with posttraumatic stress disorder^96^, a condition that is more prevalent in women who experienced childhood maltreatment^97^. Along these lines, it is also worth noting that ELA predisposes women to premenstrual dysphoria, which often includes elevated anxiety^17,18,21,86^.

Our findings suggest that theta power is elevated in MSEW mice during both diestrus and after OVX, coincident with behavioral effects suggesting altered stress-dependent activity levels and reduced grooming. Given that increased theta power was observed in MSEW mice when they are immobile, it is unlikely that the increased locomotion is driving the increased theta power, but does not preclude the possibility that increased theta power contributes to increased locomotion. Further support comes from a number of studies demonstrating that increased theta power is only tightly coupled to locomotion speed in dHIP and not vHIP^98–100^. vHIP theta power has been causally linked to increased avoidance behavior^41^. Our findings suggest that this may be reflected in other behavioral effects, such as altered climbing and grooming. In this latter regard, it may be relevant that previous studies have shown a negative correlation between grooming and theta power^99–101^. Taken together, these findings suggest that increased vHIP theta power may be contributing to the increased avoidance behavior and the reduced locomotion and grooming in potentially threatening environments (e.g., the EPM and center of the open field), as well as the increased movement in low-threat environments (e.g., the periphery of the open field and the home cage, perhaps akin to restlessness observed in humans with anxiety disorders^32,33^). It should be noted that a previous study suggested that ELA effects on grooming may be evidence of diminished “self-care”, reflecting a “depressive-like” state^59^. While it is not possible to know whether this was the case with the MSEW-induced diminished grooming we observed or whether it reflects behavioral inhibition in certain environments, it is likely relevant that there is a high comorbidity between major depressive disorder and anxiety disorders in humans^102^.

In previous work, we found that MSEW increases avoidance behavior and vHIP theta power in adult male mice^36^. The present study shows similar effects in females when they are in diestrus, but not in estrus. Protection against an MSEW-induced increase in avoidance during estrus may prevent maladaptive behavior during a stage of estrous when mice are sexually receptive. Our findings that MSEW effects on avoidance behavior in females involve progesterone signaling raise questions about whether sex differences exist in the mechanisms underlying MSEW-induced increases in avoidance behavior. Numerous studies have shown sex differences in stress effects on brain function^59,103–105^, including evidence that the hippocampus plays a critical role in determining differential stress-induced outcomes in males and females^61^. Additional studies have shown that even in cases where stress outcomes are similar in males and females, underlying mechanisms may differ^106^. Although additional work is needed to thoroughly understand how MSEW increases avoidance behavior in males and females, accumulated evidence suggests similar underlying mechanisms with overlying modulation by the estrous cycle in females. In addition to behavioral and vHIP electrophysiological effects of MSEW, we have found some sex similarities in effects on vHIP PNNs, and previous studies have shown that progesterone metabolites can reduce avoidance behavior in males^107,108^. Future work will be necessary to explore causal links among PNNs, progesterone signaling, and MSEW-induced avoidance behavior in both males and females.

Here we showed that MSEW increases avoidance behavior in females only during the diestrus phase of the estrous cycle, and that this change in behavior is associated with increased theta oscillation power in vHIP as well as alterations in the intensity and composition of vHIP perineuronal nets, which have been linked to reduced plasticity. We also showed that increased avoidance behavior is linked to MSEW-induced decreases in conversion of progesterone to the neurosteroid allopregnanolone in vHIP. Mice subjected to MSEW have higher ratios of progesterone:allopregnanolone as well as reduced expression of 5α-reductase, an enzyme important for the conversion of progesterone to allopregnanolone. We also showed that blocking progesterone receptors both systemically and in vHIP prevented increased avoidance in diestrus MSEW mice, while inhibiting allopregnanolone increased avoidance in diestrus controls. Lastly, we demonstrate that inhibition of allopregnanolone in control diestrus mice produces rapid changes in perineuronal net composition that mimic those observed in MSEW diestrus mice. Taken together, these findings identify a neuroendocrine mechanism underlying estrous cycle-induced variations in the effects of MSEW on avoidance behavior, and suggest the possibility that modulation of avoidance behavior and vHIP theta oscillations may involve neurosteroid-induced alterations in perineuronal net composition.

## Methods

### Animals and MSEW paradigm

Animal procedures were approved by the Princeton University Institutional Animal Care and Use Committee and were in accordance with the National Research Council Guide for the Care and Use of Laboratory Animals (2011). Adult male and female C57BL/6 J mice (strain#: 000664) were obtained from the Jackson Laboratory and bred onsite at the Princeton Neuroscience Institute. On the day after birth, C57BL/6 J pups were cross-fostered and placed into control or MSEW litters. MSEW included maternal separation for 4 hours daily from P2-P5, maternal separation for 8 hours from P6-P16, and weaning at P17^34–36^. Control litters were left undisturbed during this time and were weaned at P21. During maternal separation, the dam was removed from the home cage and kept in the animal holding room in a clean cage with unlimited access to food and water. The home cage containing pups was moved to an adjacent room and placed on top of a heating pad maintained at 34 °C. After weaning at P17 for MSEW mice and P21 for control mice, pups remained with their same sex littermates until behavioral testing in adulthood or surgery and behavioral testing in adulthood. Because the goal of this experiment was to explore effects of the estrous cycle on MSEW outcomes, only female offspring were used.

### Vaginal lavage and behavioral analyses

Control and MSEW mice were subjected to daily vaginal lavage beginning between 2-6 months of age to identify and track the estrous cycle of the mouse^109^ before undergoing behavioral testing. Mice were only included in an estrous cycle study after they were observed to be cycling regularly through at least two cycles. For the first study (Fig. 1), mice were examined in all four stages of the estrous cycle (proestrus, estrus, metestrus, diestrus). Thereafter, mice were selected for testing or perfusion when they were in estrus or diestrus.

### EPM testing

To measure avoidance behavior, the elevated plus maze (EPM) was used where avoidance of the open arms is considered to be evidence of avoidance behavior. Because repeated exposure to the EPM can result in habituation, we modified the testing apparatus to make the open arms more aversive by spraying them with water and increasing the brightness of the lamps (600 lux) over what we have typically used (200 lux)^36^. Pilot studies in our lab showed that the wet EPM produces more avoidance of the open arms than the dry EPM, and does not show a change in behavior with repeated testing nor any change in entries to the closed arms (Fig. S1a-d). To avoid order effects, we counterbalanced exposure to the EPM across estrous cycle stages. On the day of testing, each mouse is placed into the center of the maze and their behavior was videotaped for 5 min. To control for diurnal variations in levels of progesterone and other signaling molecules^110,111^, mice were tested during the same time of day and always during the dark phase. Time spent in open and closed arms, as well as number of entries into the arms, was determined by trained investigators watching coded videotapes so that the stage of estrous or MSEW status remained unknown. Time in the open and closed arms was scored when all four paws were present in a given arm. Entries into the open and closed arms were scored when at least two paws were present in a given arm.

### Physical activity and stress-related behaviors

Locomotion and other stress-sensitive behaviors were measured in the home cage and two distinct open field boxes in separate groups of female mice with electrodes implanted in vCA1. The open field boxes differed in translucence (opaque and clear) and in overhead lighting (lit on one or two sides). Exposure to open field environments was counterbalanced across estrous stage to avoid habituation. Mice underwent ten-minute testing in each of the conditions when in estrus or diestrus. Locomotion was measured using scores of time spent engaged in locomotion. The home cage had two elevated surfaces on opposite ends, with one of the sides having two levels. Climbing bouts onto any of the three levels were recorded as an additional measure of physical activity. Grooming, a stress-sensitive behavior^59^, was only measured in the open field, due to the low lighting of the home cage.

### Electrode implantation

Control and MSEW female mice were anesthetized and stereotaxically implanted with a customized 5-wire electrode array (Microprobes) into the unilateral vCA1 (AP: −3.5, ML: 3.4, DV: −3.5). A burr hole was drilled into the skull directly above the target region. Four additional grooves were made for implantation of surgical screws. Two of the surgical screws were implanted above the olfactory bulb and two were implanted above the cerebellum. After the screws were secured in place, the electrode was slowly lowered into the brain until it reached the target region. The ground wire was tightly wrapped around the ipsilateral caudal screw and then covered in metallic paint. After allowing the paint to dry, the electrode and ground screws were sealed in place using surgical adhesive (Metabond) and allowed to dry. Mice were singly housed after surgery to prevent cage mates from grooming each other’s head stages. Each mouse spent the same amount of time in single housing (two weeks) prior to behavioral testing and electrophysiological recording.

### Cannula implantation

Female control and MSEW mice were anesthetized and stereotaxically implanted with cannula (Plastics One) into the bilateral vCA1 (AP: −3.5, ML: 3.5, −3.5). Briefly, two burr holes were drilled directly above the target region and four additional minor grooves were drilled to hold surgical screws (two grooves over the olfactory bulb, two grooves over cerebellum). After screws were implanted, the cannula were slowly lowered to the target region. Cannula and screws were sealed in place using surgical adhesive (Metabond). Animals were group housed and allowed to recover from surgery over the course of two weeks.

### Ovariectomy and progesterone/neurosteroid pharmacological manipulations

After testing in diestrus and estrus, electrode-implanted control and MSEW mice were subjected to bilateral ovariectomy^112^. Mice were anesthetized and the ovaries were located through a single midline incision on the dorsal surface. The uterine horn and vessels were ligated and the ovaries were removed. Mice were allowed to recover for 5 days before undergoing electrophysiological recordings and behavioral testing. Ovariectomy was confirmed by examining excised ovaries and examining the body cavity after perfusion. Three additional groups of mice were ovariectomized or sham operated in order to determine hormone levels at the 5-day post-surgery period.

For the systemic pharmacology experiment, control and MSEW diestrus mice were administered vehicle, asoprisnil, or sepranolone via IP injection 30 minutes prior to testing on the wet EPM and the open field. Drug administration was counterbalanced across mice. Drugs were formulated with the following concentrations: asoprisnil 0.5 mg/kg; sepranolone 2.0 mg/kg. Compounds were dissolved in DMSO before being added to saline. The infused solutions contained 1% of the DMSO/compound solution in saline. For vehicle trials, mice received 1% DMSO in saline with no added compounds. Mice spent five minutes on the EPM and 10 minutes in the open field. Testing on the wet EPM always occurred first and preceded open field testing by roughly 10 minutes. At the conclusion of behavioral testing with a given compound, mice were excluded from additional testing until they had finished a complete estrous cycle and returned to diestrus.

For the cannula pharmacology experiment, control and MSEW diestrus mice received infusions of vehicle, asoprisnil, or sepranolone directly into vHIP 30 minutes prior to testing on the wet EPM and the open field. Drug administration was counterbalanced across mice. Mice were anesthetized using a low dose of isoflurane throughout the infusion. A 1 μL infusion volume was bilaterally infused at a rate of 500 nL/min. Infusions contained the following concentrations: asoprisnil: 0.0001 mg/μL; sepranolone: 0.0004 mg/μL. Compounds were dissolved in DMSO and added to saline, such that the infusion contained 1% of the DMSO/compound solution (i.e., 0.0001 mg asoprisnil dissolved in 0.01 μL DMSO added to 0.99 μL saline). For vehicle infusions, a 1% DMSO in saline solution was used with no added compounds. Mice spent five minutes on the EPM and 10 minutes in the open field. Testing on the wet EPM always occurred first and preceded open field testing by roughly 10 minutes. After behavioral testing with a given drug, mice were excluded from additional testing until they had finished a complete estrous cycle and returned to diestrus.

### Steroid assay

To analyze progesterone and allopregnanolone in serum and vHIP, we used modified versions of previously published methods^113,114^. Mice were briefly anesthetized (approximately 90 sec) with isoflurane and were rapidly decapitated. For cycling animals, control and MSEW animals were euthanized during estrus and diestrus. For the OVX steroid assay, the following groups were used: control diestrus mice that underwent a sham OVX operation, control OVX mice, and control OVX mice that spent 10 minutes in a brightly lit open field immediately prior to extraction. For the OVX animals, all surgeries were performed 5 days prior to sample collection. Blood was collected and centrifuged at 8000 *g* for 15 min at 4 °C. Serum was collected in a 1.5 mL Eppendorf tube and stored at −80 °C. The volume of serum was recorded for data normalization. The average serum volume was 258 μL. For vHIP extractions, brains were removed from the skull and transferred to a dissection block placed on ice. The brain was cut down the midline and the diencephalon of both hemispheres was removed to expose the hippocampus. The hippocampus was carefully extracted, and vHIP was isolated by removing 2 mm of the anterior region. The extracted vHIP was then weighed and transferred to a dounce homogenizer containing 1.5 mL of ice cold PBS. The average tissue weight was 19.73 mg. Tissue underwent 7 plunges in the homogenizer before being transferred to a 1.5 mL Eppendorf tube. Samples were kept on ice and briefly underwent sonication. Samples were then stored at −80 °C until analysis.

To extract steroids, samples were removed from the −80 °C freezer and allowed to come to room temperature. Samples were transferred to glass tubes and were suspended in a 3:1 ratio of diethyl ether (diethyl ether:sample). Samples were vortexed thoroughly for 2 minutes and then were set aside for 5 minutes. The steroid-containing diethyl ether layer was collected and transferred to a separate tube. The process was repeated two more times. The steroid-containing diethyl ether was then evaporated under a stream of nitrogen gas. Evaporated steroid samples were stored at −20 °C until analyzed. For final analysis, evaporated steroid samples were reconstituted in 0.6 mL of assay buffer (Arbor Assays) and then carefully pipetted into the appropriate ELISA kits (Progesterone: Arbor Assays (K025-H1); Allopregnanolone: Arbor Assays (K061-H1)). ELISA kits were read using a colorimeter (Molecular Devices) and analyzed with Softmax Pro 4.8 software (Molecular Devices). Steroid concentrations were normalized to account for differences in serum volume and tissue weight across samples. For normalization, serum steroid concentrations were divided by serum volume (average serum volume: 258 μL), while vHIP concentrations were divided by vHIP weight (average vHIP weight: 19.73 mg). Standard curves were unmodified for all ELISA kits.

### Electrophysiology

Local field potentials (LFPs) were recorded while mice were in the home cage and a brightly lit open field using a wireless head stage (TBSI, Harvard Biosciences), in order to minimize stress during recording. Mice were tested during diestrus and estrus, with stage of estrous counterbalanced with order of testing in the novel environment and home cage. Control and MSEW mice were habituated to wearing the headstage in the home cage for 10 minutes a day for 5 consecutive days. After habituation, mice underwent LFP recordings during a 10-minute period in the home cage and a 10-minute period in a brightly lit open field testing apparatus. LFPs were sent to a TBSI wireless 5-channel recording system, while mouse behavior was videotaped. The neural data were transmitted to a wireless receiver (Triangle Biosystems) and recorded using NeuroWare software (Triangle Biosystems). Continuous LFP data were highpass filtered at 1 Hz and notched at 60 Hz. All recordings referenced a silver wire wrapped around a ground screw implanted in the posterior parietal bone opposite of the electrode. Recordings were analyzed using NeuroExplorer software (version 5.2.1). To determine whether differences in neuronal oscillations were related to bouts of movement, separate analyses were performed on mice during periods of locomotion and immobility. To normalize LFP data, the sum of power spectra values from 0 to 100 Hz was set to equal 1.

### Histology

Mice were anesthetized with Euthasol in estrus or diestrus and transcardially perfused with 4% paraformaldehyde. To avoid potential diurnal fluctuations in PNNs^64^ and steroid levels^111^, all mice were perfused at the same time of day. Cryoprotected and frozen brain tissue was cut on a cryostat (Leica Biosystems) at 40 μm and a 1:6 series of sections was collected through vHIP. Sections were pre-blocked in PBS containing 0.3% Triton X-100 and 3% normal donkey serum for 1.5 hr at room temperature. Sections were then incubated in biotin-conjugated *Wisteria floribunda* agglutinin (WFA, 1:1000, Sigma-Aldrich), mouse anti-PV (1:500, Sigma), rabbit anti-aggrecan (1:1000, Millipore), mouse anti-C6S (1:500, Sigma), mouse anti-C4S (1:500, Amsbio), rabbit anti-proCCK (1:500, Cosmo Bio), mouse anti-SRD5A1 (1:500, Proteintech), or rabbit anti-SRD5A2 (1:200, Invitrogen) for 24 h at 4 °C, washed and then incubated in streptavidin Alexa Fluor 488 (1:1000, Invitrogen) or streptavidin Alexa Fluor 647 (1:1000), and secondary antibodies consisting of donkey anti-mouse Alexa Fluor 568 (1:500, Invitrogen), donkey anti-rabbit Alexa Fluor 488 (1:500 or 1:1000, Invitrogen), or donkey anti-mouse 568 (1:500, Invitrogen) for 1.5 h at room temperature. Washed sections were then counterstained with Hoechst 33342 (1:5,000 Molecular Probes), mounted onto slides, and coverslipped over Vectashield (Vector Laboratories). See Table S3 for information about reagents. Slides were coded until the completion of the data analysis.

### Confocal microscopy and analyses

Z-stack images from vCA1 and vDG were taken with a Leica confocal microscope and the following analyses were carried out.

### Cell density measurements

For the analysis of cell densities during estrus and diestrus, and OVX, in control and MSEW brains, neuroanatomically matched sections for each subregion were selected and the number of single and double-labelled WFA and aggrecan stained cells, WFA and C4S double labeled cells, and single and double labelled WFA, PV or pro-CCK stained cells were counted on image stacks in ImageJ (NIH). Areas were measured using Image J software. Cell densities were determined for each mouse by dividing the total number of labeled cells by the volume for that subregion (area of subregion multiplied by 40 to account for thickness of cut section).

### Optical intensity, WFA and PV cell body area measurements

For the analysis of WFA and PV intensities, as well as PNN and cell body areas in control and MSEW diestrus brains, two neuroanatomically matched sections were selected for each subregion for analysis. Z-stack 2 μm optical images of WFA + PV + double labelled cells were analyzed in ImageJ (NIH). Prior to measuring intensity, the background was subtracted (rolling ball radius = 50 pixels). Using the ROI function, a perimeter was drawn around every individual WFA cell within that subregion, including the cell body and proximal dendrites. For each WFA cell, the maximum intensity value was calculated by multiplying the maximum mean gray value by the percent area. The maximum intensities of WFA and PV were averaged per section per animal. WFA intensity values from each cell included in the analysis were separated into two bins (</> 4000 arbitrary units) and analyzed. For one experiment, control diestrus mice received an IP injection of either vehicle or sepranolone and were perfused one hour later. The concentration of the sepranolone injection was 2.0 mg/kg. vHIP WFA + C4S + cell density was subsequently analyzed.

### Optical intensity measurements

Z-stack images of vCA1 were collected using a Leica SP8 confocal with LAS X software (version 3.5.6) and a 40x oil objective. Settings remained constant throughout imaging. Collected SRD5A1 and SRD5A2 z-stack images were analyzed for optical intensity in Image J (NIH). A background subtraction using a rolling ball radius (50 pixels) was applied to the image stacks. A region of interest (ROI) was drawn around the pyramidal cell layer and the mean gray value was collected throughout the image stack.

### Statistical analyses

Data were presented as the mean + standard error of the mean (SEM). For all tests, a *p*-value of less than 0.05 was considered significant. Prior to statistical analyses, datasets were analyzed for normality and homogeneity of variance to determine whether the assumptions of parametric tests were met. For data sets that met the criteria for parametric statistics, analysis of differences between groups was determined using two-tailed unpaired t-tests, one-way ANOVA followed by Tukey post hoc tests, two-way ANOVA or mixed-effects model followed by Šidák post hoc tests. For data sets that did not meet parametric requirements, Kruskal-Wallis test and Dunn’s post hoc tests were used. A repeated measures design was used whenever possible. Behavioral data were collected and organized in Microsoft Excel (version 16.38). Graphs were produced using GraphPad Prism (version 9.3.1).

### Reporting summary

Further information on research design is available in the Nature Portfolio Reporting Summary linked to this article.

## Supplementary information

Supplementary Information

Reporting Summary

## Data Availability

The raw electrophysiology data generated in this study are available in the figshare database at https://figshare.com/articles/dataset/vCA1_electrophysiology_Estrus_Diestrus_OVX_/21534210/1. Behavioral and histological data are available from the corresponding author upon request. Source data are provided with this paper.

## Source data

Source Data
