# Author Correction: The estrous cycle modulates early-life adversity effects on mouse avoidance behavior through progesterone signaling

**DOI:** 10.1038/s41467-023-39155-4

**Published:** 2023-06-08

**Authors:** Blake J. Laham, Sahana S. Murthy, Monica Hanani, Mona Clappier, Sydney Boyer, Betsy Vasquez, Elizabeth Gould

**Affiliations:** Princeton Neuroscience Institute, Princeton, NJ 08450 USA

**Keywords:** Limbic system, Stress and resilience

Correction to: *Nature Communications* 10.1038/s41467-022-35068-w, published online 07 December 2022

The original version of the Source Data file associated with this Article included errors in the data shown for Figure 1h.

The Source Data for Fig 1h included incorrect data entries located in the first tab, rows 82–103. The incorrect data entries have been removed and replaced with the correct data entries.

The HTML has been updated to include a corrected version of the Source Data file; the original incorrect version of the Source Data file can be found as Supplementary Information associated with this Correction.

## Supplementary information

Original Source Data

Updated Source Data

