# Supplementary material for: The estrous cycle modulates early-life adversity effects on mouse avoidance behavior through progesterone signaling
Source: Nat Commun. 2022 Dec 7;13:7537. doi: 10.1038/s41467-022-35068-w (PMC9729614; doi:10.1038/s41467-022-35068-w)
Supplement: Supplementary file 1 — Supplementary Information [file 41467_2022_35068_MOESM1_ESM.pdf]

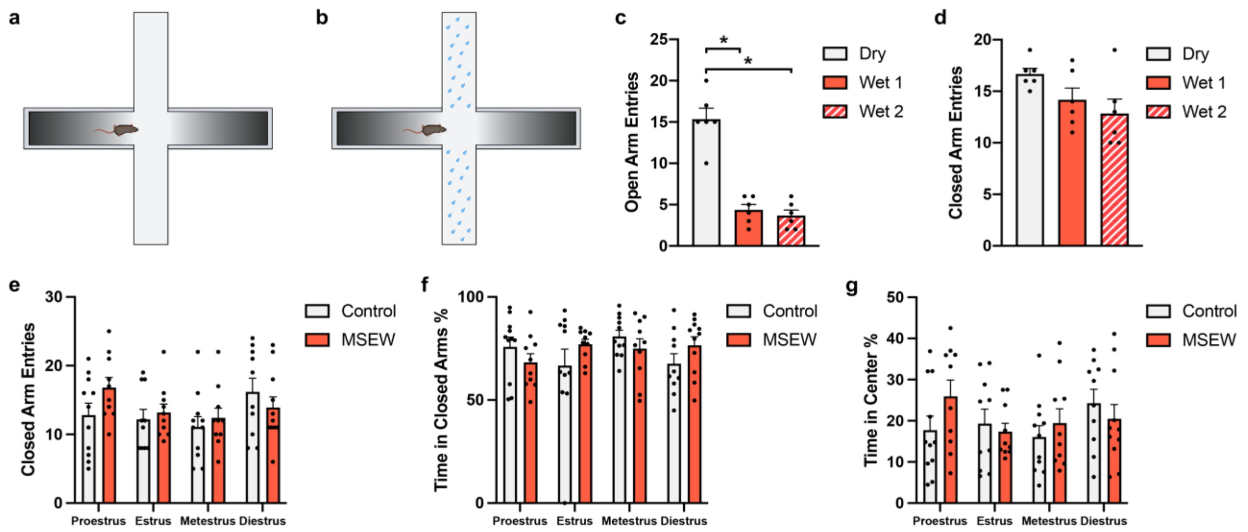

Figure S1
Avoidance behavior is increased on the modified "wet" EPM compared to the traditional "dry" EPM with no change after second exposure

Schematics of the **a** traditional and **b** modified EPM showing water droplets on open arms. **c** Open arm entries are lower when exposed to the wet EPM compared to the dry EPM, with no difference between the first and second exposure to the wet EPM ($F_{2,15} = 48.29$, $p = 0.0001$; dry-wet 1 $p = 0.0001$, dry-wet 2 $p = 0.0001$; wet 1-wet 2 $p = 0.8724$; $n = 6$). **d** Closed arm entries are not different when exposed to the dry or wet EPM ($n = 6$). **e** No difference in closed arm entries when mice are tested according to estrous stage (Control $n = 11$, MSEW $n = 11$). **f** No difference in percent time in closed arms when mice are tested according to estrous stage (Control $n = 11$, MSEW $n = 11$). **g** No difference in percent time spent in center of wet EPM when mice are tested according to estrous stage (Control $n = 11$, MSEW $n = 11$). MSEW = maternal separation early weaning. *$p < 0.05$; one-way repeated measures ANOVA with Tukey tests (**c**,**d**); two-way repeated measures ANOVA with Šidák tests (**e**,**f**,**g**). Data are presented as mean values + SEM (**c-g**). Images in **a,b** were created using BioRender.com. Source data are provided as a Source Data file.

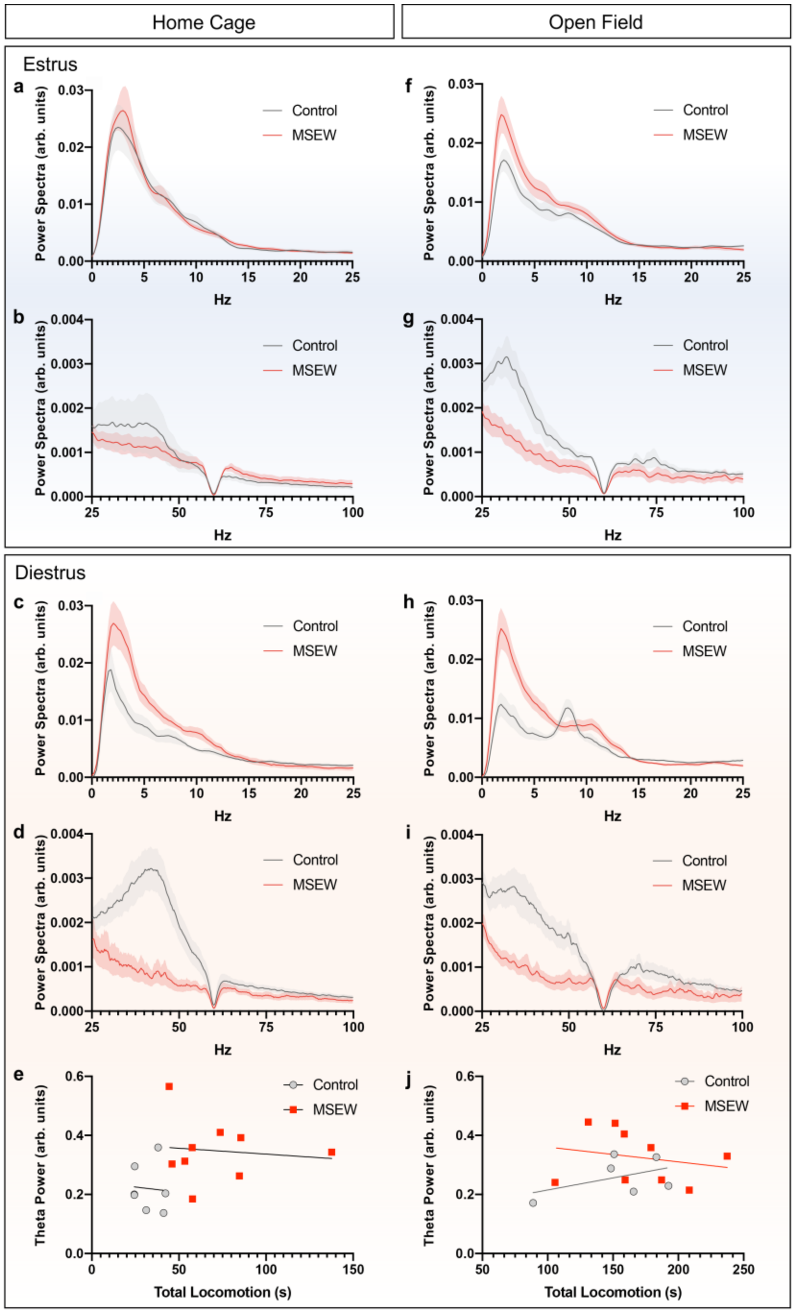

Figure S2
Estrous cycle MSEW and control vCA1 theta and gamma in the home cage and open field

a,**b** vCA1 power spectra in home cage during estrus. **c**,**d** vCA1 power spectra in home cage during diestrus. **e** Graph showing lack of correlation between theta power and locomotion duration in home cage diestrus animals (Control *n* = 6, MSEW *n* = 9). **f**,**g** vCA1 power spectra in open field during estrus. **h**,**i** vCA1 power spectra in open field during diestrus. **j** Graph showing lack of correlation between theta power and locomotion duration in open field diestrus animals (Control *n* = 6, MSEW *n* = 9). Arb. units = arbitrary units; MSEW = maternal separation early weaning. Data are presented as mean values ± SEM error bands for **a**-**d** and **f**-**i**. Source data are provided as a Source Data file.

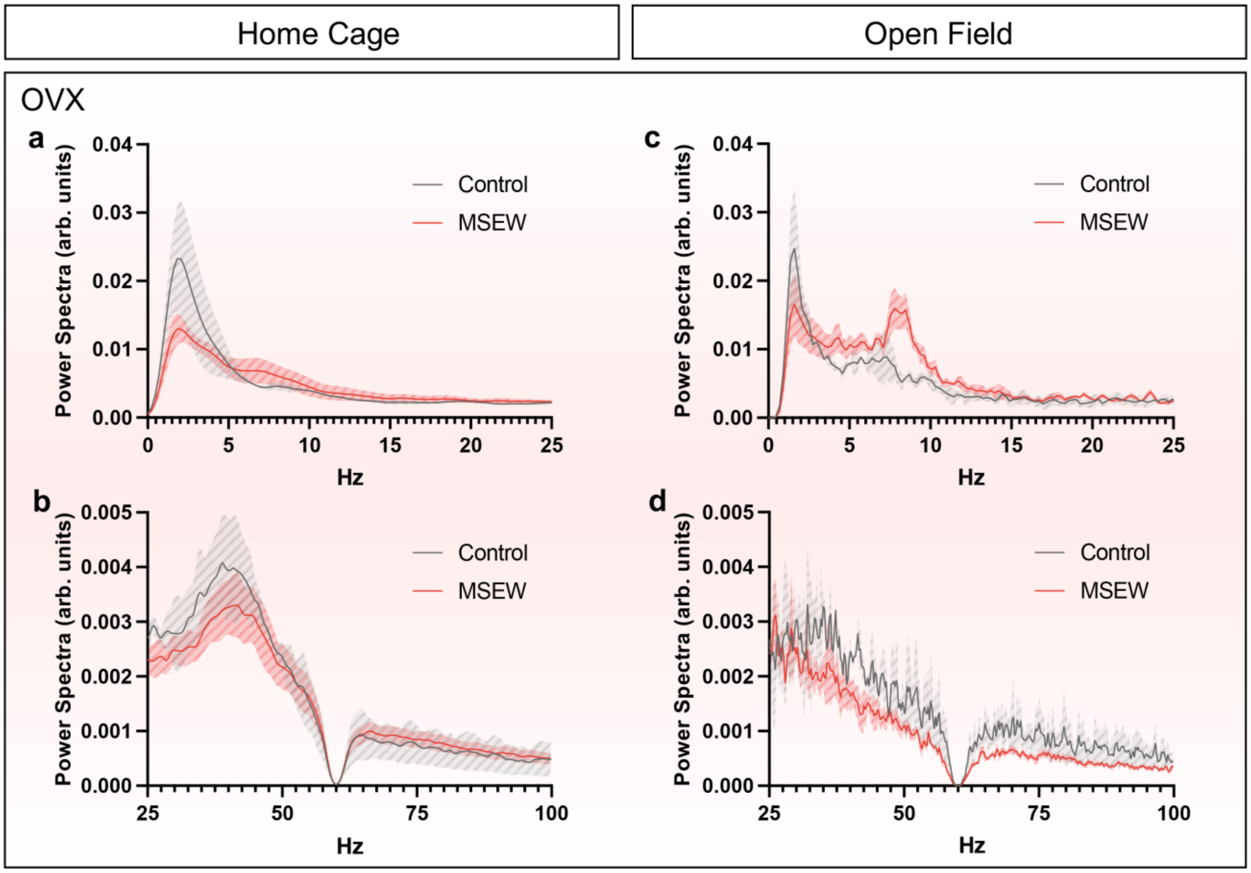

Figure S3
OVX MSEW and control vCA1 theta and gamma in the home cage and open field

a,**b** vCA1 power spectra in home cage after OVX (Control *n* = 2, MSEW *n* = 4). **c**,**d** vCA1 power spectra in open field after OVX (Control *n* = 2, MSEW *n* = 4). Arb. units=arbitrary units; MSEW = maternal separation early weaning; OVX = ovariectomy. Data are presented as mean values ± SEM for error bands (**a-d**). Source data are provided as a Source Data file.

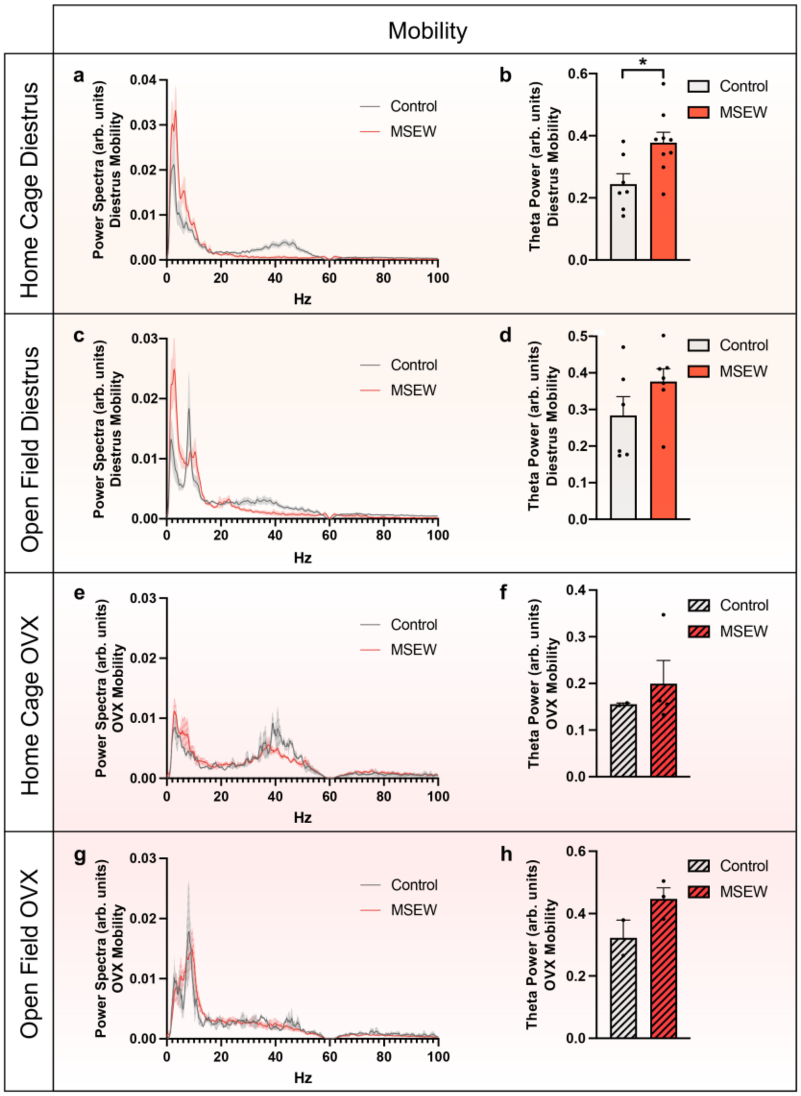

Figure S4
Estrous cycle and OVX control and MSEW vCA1 theta power in home cage and open field during mobility

a Diestrus vCA1 power spectra during mobility in home cage. **b** Diestrus vCA1 theta power during mobility in home cage is higher in MSEW than control (t_{14} = 2.777, p = 0.0148; Control n = 7, MSEW n = 9). **c** Diestrus vCA1 power spectra during mobility in open field. **d** Diestrus vCA1 theta power during mobility in open field (Control n = 6, MSEW n = 7). **e** OVX vCA1 power spectra during mobility in home cage. **f** OVX vCA1 theta power during mobility in home cage (Control n = 2, MSEW n = 4). **g** OVX vCA1 power spectra during mobility in home cage. **h** OVX vCA1 theta power during mobility in home cage (Control n = 2, MSEW n = 4). Arb. units = arbitrary units; MSEW = maternal separation early weaning; OVX = ovariectomy. *$p < 0.05$, two-sided unpaired t-tests (**b**,**d**,**f**,**h**). Data are presented as mean values + SEM for error bars (**b,d,f,h**); ± SEM for error bands (**a,c,e,g**). Source data are provided as a Source Data file.

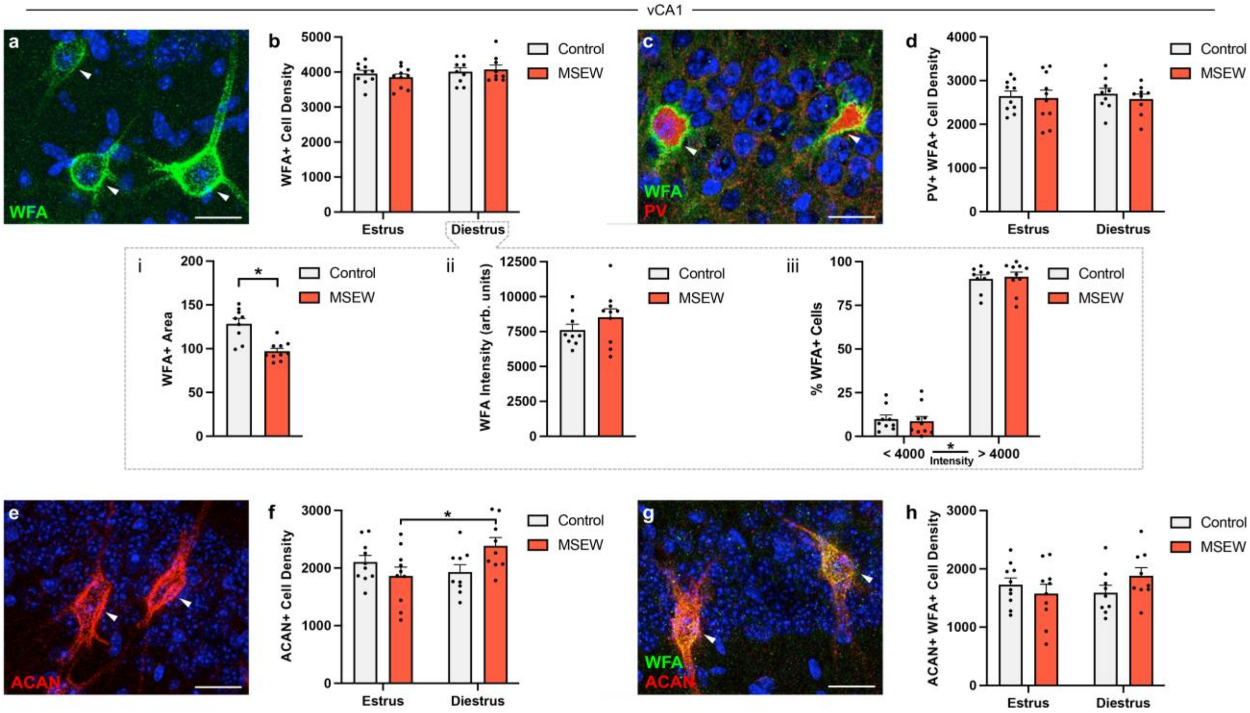

Figure S5
MSEW influences PNN size in vCA1

a Confocal example of WFA+ cells (green) in vCA1. **b** There is no difference in the density of cells surrounded by WFA+ PNNs in the vCA1 across estrous (Control: Estrus *n* = 10, Diestrus *n* = 9; MSEW: Estrus *n* = 10, Diestrus: *n* = 9). **bi** During diestrus, MSEW mice have PNNs with smaller cross-sectional areas than control mice in vCA1 (t_{17} = 4.636, *p* = 0.0002; Control *n* = 9, MSEW *n* = 10). **bii** During diestrus, there is no difference in WFA+ intensity between control and MSEW mice in vCA1 (Control *n* = 7, MSEW *n* = 10). **biii** During diestrus, control and MSEW vCA1 WFA+ PNNs are primarily in high intensity bins (Control *n* = 9, MSEW *n* = 10). **c** Confocal example of PV+ (red) WFA+ (green) cells in vCA1. **d** The density of PV+ cells surrounded by WFA+ PNNs in the vDG gyrus remains unchanged across the estrous cycle (Control: Estrus *n* = 10, Diestrus *n* = 9; MSEW: Estrus *n* = 10, Diestrus: *n* = 9). **e** Confocal example ACAN+ cells (red) in vDG. **f** The density of cells expressing ACAN in vDG changes across the estrous cycle ($F_{1,34}$ = 6.534, *p* = 0.0152; Estrus-Diestrus MSEW *p* = 0.0209; Control: Estrus *n* = 10, Diestrus *n* = 9; MSEW: Estrus *n* = 10, Diestrus: *n* = 9). **g** Confocal example of ACAN+ (green) WFA+ (red) cells in vDG. **h** The density of ACAN+WFA+ cells in vDG changes across the estrous cycle (Control: Estrus *n* = 10, Diestrus *n* = 9; MSEW: Estrus *n* = 10, Diestrus: *n* = 9). ACAN = aggrecan; arb. units = arbitrary units; MSEW = maternal separation early weaning; PV = parvalbumin; vCA1 = ventral CA1. **p* < 0.05 two-way ANOVA (Estrous x MSEW) with Šidák tests (**b**,**biii**,**d**,**f**,**h**); two-sided unpaired *t*-tests (**bi**,**bii**). Scale bars for **a**,**c**,**e**,**g** represent 20 μm. Data are presented as mean values + SEM (**b,bi,bii,biii,d,f,h**). Source data are provided as a Source Data file.

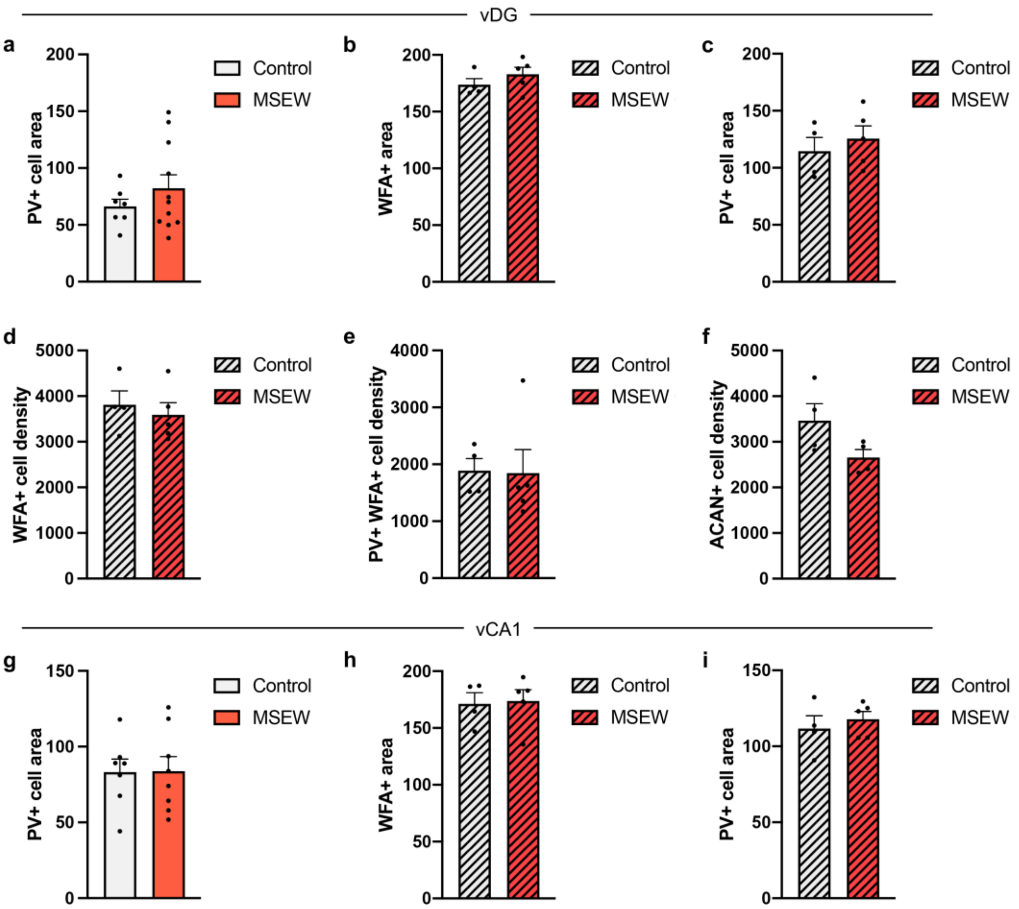

Figure S6
MSEW does not alter PNN size or number in OVX mice

a No differences were observed between control and MSEW during diestrus in vDG PV+ cell body area (Control *n* = 7, MSEW *n* = 11), **b** OVX WFA+ area (Control *n* = 4, MSEW *n* = 5), **c** OVX PV+ cell body area (Control *n* = 4, MSEW *n* = 5), **d** OVX WFA+ density (Control *n* = 4, MSEW *n* = 5), **e** OVX PV+WFA+ density (Control *n* = 4, MSEW *n* = 5), or **f** OVX ACAN+ density (Control *n* = 4, MSEW *n* = 5). **g** No differences were observed between control and MSEW during diestrus in vCA1 PV+ cell body area (Control *n* = 7, MSEW *n* = 8), **h** OVX WFA+ area (Control *n* = 4, MSEW *n* = 5), or **i** OVX PV cell body area (Control *n* = 4, MSEW *n* = 5). ACAN = aggrecan; MSEW = maternal separation early weaning; OVX = ovariectomy; PV = parvalbumin; vDG = ventral dentate gyrus; vCA1 = ventral CA1. Two-sided unpaired *t*-tests. Data are presented as mean values + SEM (**a-i**). Source data are provided as a Source Data file.

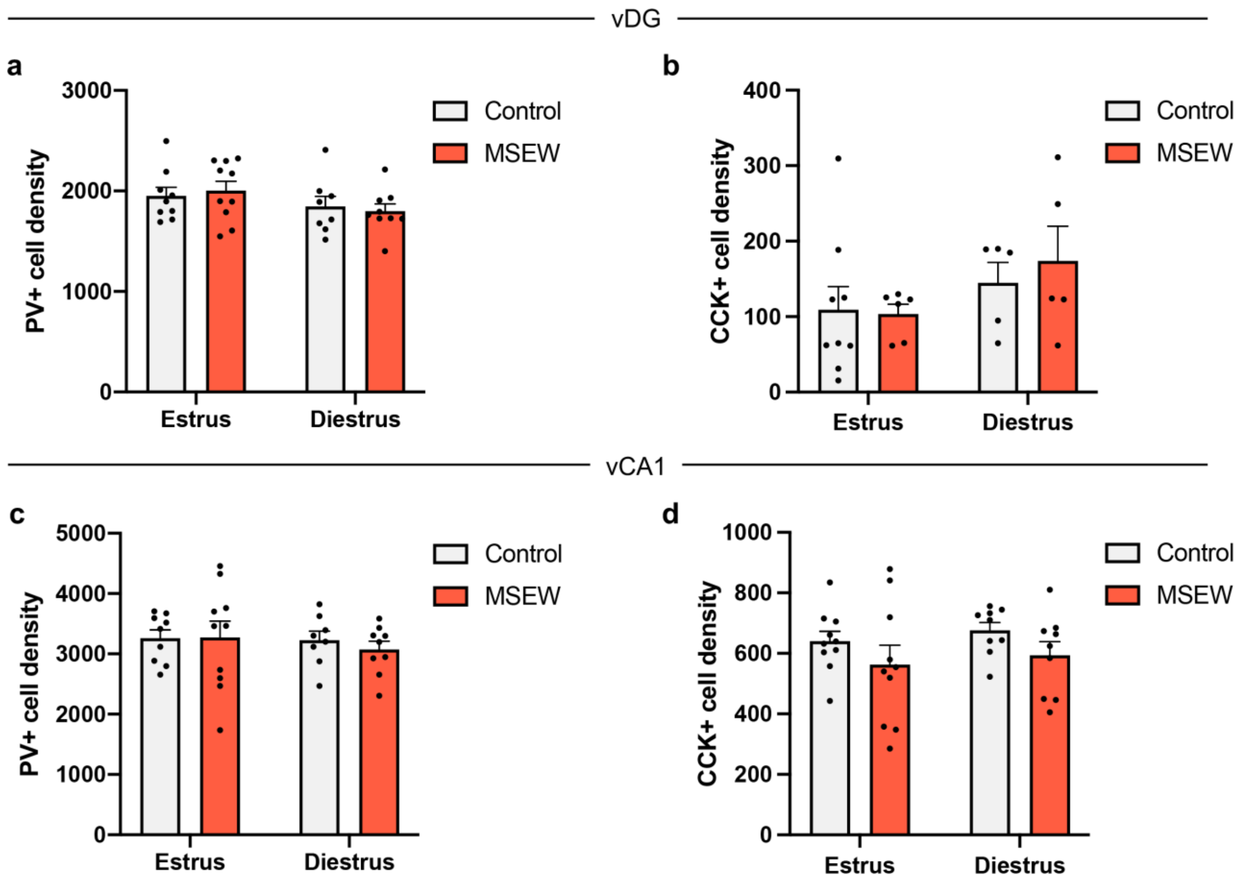

Figure S7
MSEW does not influence the expression of certain inhibitory interneuron markers in the ventral hippocampus

a There is no effect of MSEW or estrous on PV+ density in vDG (Control: Estrus *n* = 9, Diestrus *n* = 8; MSEW: Estrus *n* = 10, Diestrus: *n* = 9). **b** There is no effect of MSEW or estrous on CCK+ density in vDG (Control: Estrus *n* = 9, Diestrus *n* = 5; MSEW: Estrus *n* = 6, Diestrus: *n* = 5). **c** There is no effect of MSEW or estrous on PV+ density in vCA1 (Control: Estrus *n* = 9, Diestrus *n* = 8; MSEW: Estrus *n* = 10, Diestrus: *n* = 9). **d** There is no effect of MSEW or estrous on CCK+ density in vCA1 (Control: Estrus *n* = 10, Diestrus *n* = 9; MSEW: Estrus *n* = 10, Diestrus: *n* = 9). CCK = cholecystokinin; MSEW = maternal separation early weaning; PV = parvalbumin; vDG = ventral dentate gyrus; vCA1 = ventral CA1. Two-way ANOVA with Šidák tests. Data are presented as mean values + SEM (**a-d**). Source data are provided as a Source Data file.

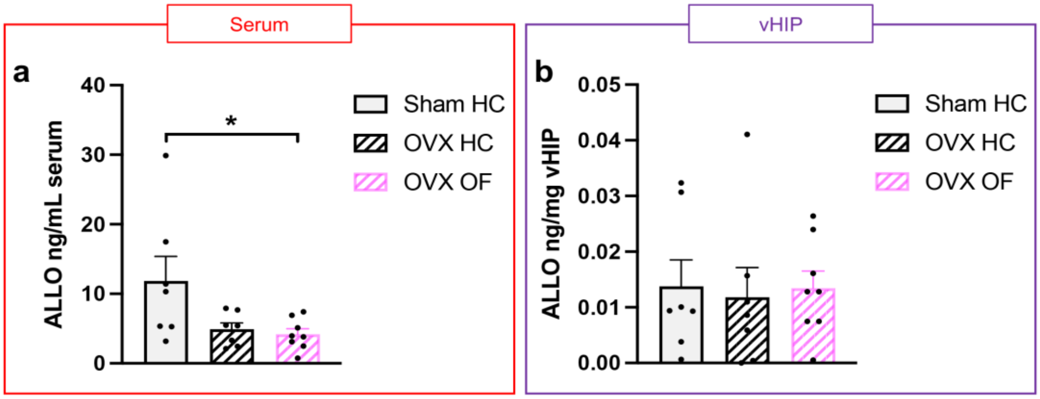

Figure S8
Serum allopregnanolone levels are decreased after OVX

a Serum allopregnanolone levels are decreased after OVX relative to sham-operated controls ($F_{2,19}$ = 4.155, p = 0.0318; Sham HC-OVX HC p = 0.0755, Sham HC-OVX OF p = 0.0396; Sham HC n = 7, OVX HC n = 7, OVX OF n = 8). **b** OVX has no effect on allopregnanolone expression in vHIP (Sham HC n = 7, OVX HC n = 7, OVX OF n = 8). ALLO = allopregnanolone; HC = home cage; OF = open field; vHIP = ventral hippocampus. *p < 0.05; one-way ANOVA with Tukey tests. Data are presented as mean values + SEM (**a,b**). Source data are provided as a Source Data file.

Supplementary Tables

Table S1:

Percentage of double labeled cells in the ventral dentate gyrus

vDG	Control Estrus	MSEW Estrus	Control Diestrus	MSEW Diestrus	Control OVX	MSEW OVX
PV+WFA+/PV$^+$	77.3±3.5	79.5±3.1	74.4±1.6	73.8±3.9	85.8±4.5	81.8±2.8
PV+WFA+/WFA+	56.8±3.1	49.0±3.6	55.5±2.5	56.7±4.6	59.7±4.7	59.8±7.2
ACAN$^+$WFA$^+$/ACAN$^+$	57.9±2.5	60.0±2.4	55.6±2.3	58.1±3.3	62.9±2.0	69.4±5.6
ACAN$^+$WFA$^+$/WFA$^+$	53.9±2.3	52.2±2.9	45.8±2.0	46.6±2.7	63.8±1.1	65.8±4.5

No significant differences were observed between control estrus and diestrus, or between control and MSEW within estrous stage or with OVX.

Table S2: Percentage of double labeled cells in the ventral CA1 region

vCAI	Control Estrus	MSEW Estrus	Control Diestrus	MSEW Diestrus	Control OVX	MSEW OVX
PV+WFA+/PV$^+$	82.0±1.1	76.2±3.5	82.7±2.3	83.9±1.2	84.1±3.1	82.3±4.1
PV+WFA+/WFA+	74.0±1.7	67.2±7.5	72.4±1.5	71.8±2.4	58.1±4.8	54.9±2.7
ACAN$^+$WFA$^+$/ACAN$^+$	81.7±2.8	84.8±1.6	81.6±1.8	80.7±2.5	93.1±1.1	89.6±2.7
ACAN$^+$WFA$^+$/WFA$^+$	51.9±1.7	49.4±3.1	47.5±1.8	51.7±2.5	69.3±3.1	65.2±7.7

No significant differences were observed between control estrus and diestrus, or between control and MSEW within estrous stage or with OVX.

Table S3: Reagents for Histochemistry

Reagent	Host	Manufacturer	Catalog number	Dilution
Anti-PV	Mouse	Sigma-Aldrich	P3088	1:500
Anti-proCCK	Rabbit	Cosmo Bio	NMD-MSFR105030	1:500
Wisteria floribunda agglutinin	-	Millipore Sigma	L1516	1:1000
Anti-Aggrecan	Rabbit	Millipore Sigma	AB1031	1:1000
Anti-Chondrotin-4-Sulfate	Mouse	Amsbio	270421-1	1:500
Anti-SRD5A1	Mouse	Proteintech	66329-1-lg	1:500
Anti-SRD5A2	Rabbit	Invitrogen	MA5-37985	1:200
