# Supplementary material for: The estrous cycle modulates early-life adversity effects on mouse avoidance behavior through progesterone signaling
Source: Nat Commun. 2022 Dec 7;13:7537. doi: 10.1038/s41467-022-35068-w (PMC9729614; doi:10.1038/s41467-022-35068-w)
Supplement: Supplementary file 2 — Reporting Summary [file 41467_2022_35068_MOESM2_ESM.pdf]

nature portfolio

Corresponding author(s): Elizabeth Gould

Last updated by author(s): Nov 10, 2022

Reporting Summary

Nature Portfolio wishes to improve the reproducibility of the work that we publish. This form provides structure for consistency and transparency in reporting. For further information on Nature Portfolio policies, see our Editorial Policies and the Editorial Policy Checklist.

Statistics

For all statistical analyses, confirm that the following items are present in the figure legend, table legend, main text, or Methods section.

n/a	Confirmed	
☐	☒	The exact sample size (*n*) for each experimental group/condition, given as a discrete number and unit of measurement
☐	☒	A statement on whether measurements were taken from distinct samples or whether the same sample was measured repeatedly
☐	☒	The statistical test(s) used AND whether they are one- or two-sided<br>*Only common tests should be described solely by name; describe more complex techniques in the Methods section.*
☒	☐	A description of all covariates tested
☐	☒	A description of any assumptions or corrections, such as tests of normality and adjustment for multiple comparisons
☐	☒	A full description of the statistical parameters including central tendency (e.g. means) or other basic estimates (e.g. regression coefficient) AND variation (e.g. standard deviation) or associated estimates of uncertainty (e.g. confidence intervals)
☐	☒	For null hypothesis testing, the test statistic (e.g. *F*, *t*, *r*) with confidence intervals, effect sizes, degrees of freedom and *P* value noted<br>*Give P values as exact values whenever suitable.*
☒	☐	For Bayesian analysis, information on the choice of priors and Markov chain Monte Carlo settings
☒	☐	For hierarchical and complex designs, identification of the appropriate level for tests and full reporting of outcomes
☒	☐	Estimates of effect sizes (e.g. Cohen's *d*, Pearson's *r*), indicating how they were calculated

Our web collection on statistics for biologists contains articles on many of the points above.

Software and code

Policy information about availability of computer code

Data collection	Electrophysiological recordings were analyzed using Neuroexplorer software version 5.21 (Nex Technologies).<br>Confocal images were collected using LAS X software 3.5.6<br>ImageJ (NIH) software was used for the analysis of cell densities, PNN and cell body areas, and WFA, PV, SRD5A1, and SRD5A2 optical intensities.<br>Steroid concentrations were analyzed using Softmax Pro 4.8 (Molecular Devices)<br>Data were organized using Microsoft Excel 16.38 spreadsheets .
Data analysis	GraphPad Prism (9.3.1) was used for graphic visualization and statistical analysis.

For manuscripts utilizing custom algorithms or software that are central to the research but not yet described in published literature, software must be made available to editors and reviewers. We strongly encourage code deposition in a community repository (e.g. GitHub). See the Nature Portfolio guidelines for submitting code & software for further information.

Data

Policy information about availability of data

All manuscripts must include a data availability statement. This statement should provide the following information, where applicable:

- Accession codes, unique identifiers, or web links for publicly available datasets
- A description of any restrictions on data availability
- For clinical datasets or third party data, please ensure that the statement adheres to our policy

All data generated from this study are accessible in the Supplementary Information/Source Data files. Electrophysiology data are available on Figshare.com

https://figshare.com/articles/dataset/vCA1_electrophysiology_Estrus_Diestrus_OVX_/21534210/1
Additional information will be available from the corresponding author upon request (goulde@princeton.edu).

Field-specific reporting

Please select the one below that is the best fit for your research. If you are not sure, read the appropriate sections before making your selection.

- [x] Life sciences
- [ ] Behavioural & social sciences
- [ ] Ecological, evolutionary & environmental sciences

For a reference copy of the document with all sections, see nature.com/documents/nr-reporting-summary-flat.pdf

Life sciences study design

All studies must disclose on these points even when the disclosure is negative.

Sample size	Sample sizes were determined according to the standards of the field and previous studies on avoidance behavior and ventral hippocampus performed in our laboratory (Murthy S, Kane GA, Katchur NJ, Lara Mejia PS, Obiofuma G, Buschman TJ, McEwen BS, Gould E. Perineuronal Nets, Inhibitory Interneurons, and Anxiety-Related Ventral Hippocampal Neuronal Oscillations Are Altered by Early Life Adversity. Biol Psychiatry. 2019 Jun 15;85(12):1011-1020. doi: 10.1016/j.biopsych.2019.02.021; Schoenfeld TJ, Kloth AD, Hsueh B, Runkle MB, Kane GA, Wang SS, Gould E. Gap junctions in the ventral hippocampal-medial prefrontal pathway are involved in anxiety regulation. J Neurosci. 2014 Nov 19;34(47):15679-88. doi: 10.1523/JNEUROSCI.3234-13.2014.
Data exclusions	Data exclusion criteria were determined during the experimental design stage.<br>Animals were removed from EPM analyses if they spent 0 seconds in the open arm across all trials (estrous stages; drug treatments).<br>Electrode recordings were excluded upon detection of high 60 Hz noise on the power spectra.
Replication	Experiments in Figures 1 and 51-r have internal replications but no studies were done to specifically replicate experiments.
Randomization	Pregnant females were randomly divided into control and MSEW groups before giving birth. Behavioral testing and electrophysiological recordings were performed across stages of the estrous cycle in a randomized fashion. Order of drug administration was randomized. Steroid collection from control and MSEW female mice was randomized between estrus and diestrus.
Blinding	Animal IDs were coded to prevent potential researcher bias. Experimenters were blind to the animal ID and group number until after the data analyses were completed.

Reporting for specific materials, systems and methods

We require information from authors about some types of materials, experimental systems and methods used in many studies. Here, indicate whether each material, system or method listed is relevant to your study. If you are not sure if a list item applies to your research, read the appropriate section before selecting a response.

Materials & experimental systems

n/a	Involved in the study	
☐	☒	Antibodies
☒	☐	Eukaryotic cell lines
☒	☐	Palaeontology and archaeology
☐	☒	Animals and other organisms
☒	☐	Human research participants
☒	☐	Clinical data
☒	☐	Dual use research of concern

Methods

n/a	Involved in the study	
☒	☐	ChIP-seq
☒	☐	Flow cytometry
☒	☐	MRI-based neuroimaging

Antibodies

Antibodies used	Primary antibodies:<br>Rabbit anti-aggrecan (1:1000, AB1031, Millipore)<br>Mouse anti-C4S (1:500, 270421-1, Amsbio)<br>Rabbit anti-proCCK (1:500, NMD-MSFR105030, Cosmo Bio)<br>Mouse anti-PV (1:500, p3088, Sigma)<br>Mouse anti-SRD5A1 (1:500, 66329, 1-lg, Proteintech)<br>Rabbit anti-SRD5A2 (1:200, MA5-37985, Invitrogen)<br>Secondary Antibodies:<br>All from Thermo Fisher Scientific (1:1000)

Validation	Rabbit anti-aggrecan (1:1000, AB1031, Millipore): 32 citations<br>Validation from manufacturer's datasheet: validated for Mouse WB 1:500<br>(https://www.emdmillipore.com/US/en/product/Anti-Aggrecan-Antibody,MM_NF-AB1031)<br>Mouse anti-C4S (1:500, 270421-1, Amsbio): 4 citations<br>Validation from manufacturer's datasheet: validated for chicken, bovine, rat IHC, ELISA, IP<br>(https://www.amsbio.com/ab-chondroitin-4-sulfate-purified-270421-1)<br>Rabbit anti-proCCK (1:500, NMD-MSFR105030, Cosmo Bio): 1 citation<br>Validation from manufacturer's datasheet: validated for Mouse ISH<br>(https://www.cosmobiousa.com/products/anti-procck-pab)<br>Mouse anti-PV (1:500, p3088, Sigma): 566 citations<br>Validation from manufacturer's datasheet: validated for bovine, cat, dog, fish, goat, human, pig, rabbit, rat IHC, ELISA,WB<br>(https://www.sigmaaldrich.com/US/en/product/sigma/p3088)<br>Mouse anti-SRD5A1 (1:500, 66329, 1-lg, Proteintech): 5 citations<br>Validation from manufacturer's datasheet: validated for human WB, IHC, IF, ELISA<br>(https://www.ptglab.com/products/SRD5A1-Antibody-66329-1-Ig.htm)<br>Rabbit anti-SRD5A2 (1:200, MA5-37985, Invitrogen): 0 citations<br>Validation from manufacturer's datasheet: validated for Human, Mouse, Rat WB, IHC<br>(https://www.thermofisher.com/antibody/product/SRD5A2-Antibody-clone-ARC2287-Recombinant-Monoclonal/MA5-37985)

Animals and other organisms

Policy information about studies involving animals; ARRIVE guidelines recommended for reporting animal research

Laboratory animals	C57BL/6J female mice (8-30 weeks of age) were used in experiments. Mice were housed in standard cages. All mice were housed in groups except for the electrophysiology mice, which were singly housed. Mice kept in a reverse light-dark cycle and tested in the dark. Humidity of the room was approximately 50%. The mice were provided ad lib access to food and water.
Wild animals	The study did not include wild animals.
Field-collected samples	The study did not include samples collected from the field.
Ethics oversight	Animal procedures were approved by the Princeton University Institutional Animal Care and Use Committee and were in accordance with the National Research Council Guide for the Care and Use of Laboratory Animals. Adult male and female C57BL/6J mice were obtained from The Jackson Laboratory and bred on-site at the Princeton Neuroscience Institute.

Note that full information on the approval of the study protocol must also be provided in the manuscript.
